# Supplementary material for: Cytoprotective Activity of Newly Synthesized 3-(Arylmethylamino)-6-Methyl-4-Phenylpyridin-2(1H)-Ones Derivatives
Source: Molecules. 2022 Aug 23;27(17):5362. doi: 10.3390/molecules27175362 (PMC9458246; doi:10.3390/molecules27175362)

Supplementary Materials

Cytoprotective activity of newly synthesized 3-(arylmethylamino)-6-methyl-4-phenylpyridin-2(1*H*)-ones derivatives

Shynggys Sergazy*[1,2], Zarina Shulgau[1], Aigerim Zhulikeyeva[1], Yerlan Ramankulov[1], Irina V. Palamarchuk[3] and Ivan V. Kulakov[1,3]

[1] RSE "National Center for Biotechnology", 13/5 Kurgalzhynskoe road, Nur-Sultan, Kazakhstan
[2] National Laboratory Astana, Nazarbayev University, Nur-Sultan, Kazakhstan
[3] Institute of Chemistry, Tyumen State University, 15a Perekopskaya St., Tyumen 625003, Russia
* Correspondence: shynggys.sergazy@gmail.com; Tel.: +7 702 1999758

Experimental

IR spectra were recorded on an Infralum FT-801 spectrometer for KBr pellets. ^{1}H and ^{13}C NMR spectra were recorded on a Bruker DRX400 (400 and 100 MHz, respectively) and Bruker AVANCE 500 (500 and 125 MHz, respectively) instruments using DMSO-d_6 (compound **2b, 2e-g, 3g**), DMSO-d_6 + CF_3COOH (compound **2d**) or $CDCl_3$ (remaining compounds) with TMS as internal standard. Elemental analysis was performed on a Carlo Erba 1106 CHN instrument. Melting points were determined using a Koffler hot bench. Monitoring of the reaction course and the purity of the products was carried out by TLC on Sorbfilplates and visualized using iodine vapor or UV light.

Table of Contents:

1. Synthetic procedures....................2
2. Spectroscopic and physical data 2
3. ^{1}H and ^{13}C NMR Spectra 4

Synthetic procedures

Aldehydes were commercially available and used as received, 3-aminopyridin-2(1*H*)-one **1** was synthesized according to procedure [1].

Typical procedure for the synthesis of imines 2 a-h. The mixture of 3-amino-pyridin-2(1*H*)-one **1** (200 mg, 1 mmol), aromatic aldehyde (1.2 mmol) and catalytic amount of formic acid in 5 ml *i*-propanol was refluxed for 1-3 h. After cooling the reaction mixture, precipitated imine **2a-h** was filtered off and washed with hexane.

Typical procedure for the synthesis of compounds 3 a-h. To a suspension of imine **2a-h** (1 mmol) in 2-PrOH (15 ml) were added water (3 ml) and sodium borohydride (0.380 g, 10 mmol) with stirring at a temperature of 25-35 °C; the reaction mixture was stirred for 10-15 h. Then the reaction mixture was poured into a beaker with ice-cold water (150 ml). The aqueous layer was extracted with chloroform (3×25 ml), the organic layer was dried over Na_2SO_4, the solvent was removed by distillation, and the residue was triturated with hexane. The crude product was recrystallized from a 1:2 mixture of 2-PrOH and hexane.

Spectroscopic and physical data

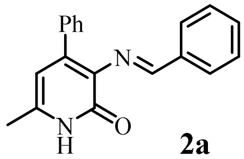<br>**2a**<br>Chemical Formula: $C_{19}H_{16}N_2O$<br>Molecular Weight: 288,3500<br>Elemental Analysis: C, 79.14; H, 5.59; N, 9.72; O, 5.55	**3-(Benzylideneamino)-6-methyl-4-phenylpyridin-2(1*H*)-one (2a)**<br>Yield: 236 mg (82%), yellow crystals, m.p.: 214-215 °C (2-propanol- chloroform).<br>IR (KBr, cm^{-1}): 1525, 1636, 3352, 3450.<br>^{1}H NMR (400 MHz, $CDCl_3$) δ: 2.41 (s, 3H), 6.25 (s, 1H), 7.35-7.40 (m, 5H), 7.48-7.53 (m, 3H), 7.75 (dd, ^{3}J = 7.1 Hz, ^{4}J = 2.4 Hz, 2H), 9.35 (s, 1H), 13.18 (br. s, 1H).<br>^{13}C NMR (100 MHz, $CDCl_3$) δ: 18.7, 108.7, 121.6, 127.5, 128.0, 128.4, 128.6, 130.0, 130.6, 131.0, 131.7, 137.7, 140.8, 146.3, 161.2, 162.6.<br>Anal. Calcd for $C_{19}H_{16}N_2O$: C, 79.14; H, 5.59; N, 9.72. Found: C, 79.52; H, 5.87; N, 9.91.
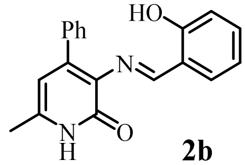<br>**2b**<br>Chemical Formula: $C_{19}H_{16}N_2O_2$<br>Molecular Weight: 304,3490<br>Elemental Analysis: C, 74.98; H, 5.30; N, 9.20; O, 10.51	**3-((2-Hydroxybenzylidene)amino)-6-methyl-4-phenylpyridin-2(1*H*)-one (2b).**<br>Yield: 289 mg (95%), yellow crystals, m.p.: 251-252 °C (2-propanol).<br>IR (KBr, cm^{-1}): 1282, 1466, 1626. 2826, 3454.<br>^{1}H NMR (400 MHz, DMSO-d6) δ: 2.24 (s, 3H, CH_3); 6.13 (s, 1H, H-5); 6.71 (d, 1H, ^{3}J = 8.2 Hz, H-3' Ar); 6.86 (td, 1H, ^{3}J = 7.4 Hz, ^{4}J = 1.0 Hz, H-5' Ar); 7.26 (td, 1H, ^{3}J = 7.7 Hz, ^{4}J = 1.5 Hz, H-4' Ar); 7.36-7.44 (m, 6H, 5H Ph, H-6' Ar); 9.86 (s, 1H, =C-H); 12.16 (s, 1H, NH); 12.59 (s, 1H, OH).<br>^{13}C NMR (100 MHz, DMSO-d6) δ: 18.3, 107.1, 116.3, 118.8, 119.7, 127.9, 128.27, 128.31, 128.5, 132.3, 132.4, 137.9, 142.8 , 147.2, 159.1, 159.9, 164.3.<br>Anal. Calcd for $C_{19}H_{16}N_2O_2$: C, 75.31; H, 5.66; N, 9.57. Found: C, 74.98; H, 5.30; N, 9.20.

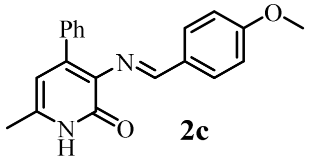<br>**2c**<br>Chemical Formula: $C_{20}H_{18}N_2O_2$<br>Molecular Weight: 318,38<br>Elemental Analysis: C, 75.45; H, 5.70; N, 8.80; O, 10.05	**3-((4-Methoxybenzylidene)amino)-6-methyl-4-phenylpyridin-2(1*H*)-one (2c)**<br>Yield: 261 mg (82%), yellow crystals, m.p.: 217-219 °C (2-propanol- chloroform).<br>IR (KBr, cm^{-1}): 1248, 1574, 1625, 2901.<br>^1H NMR (400 MHz, $CDCl_3$) δ: 2.40 (s, 3H, CH_3); 3.83 (s, 3H, OCH_3); 6.23 (d, 1H, ^4J = 0.8 Hz, H-5); 6.90 (d, 2H, ^3J = 8.8 Hz, H-3,5 Ar); 7.38 (d, 2H, ^3J = 7.4 Hz, H-2,6 Ph); 7.51 (td, 3H, ^3J = 8.7 Hz, ^4J = 1.5 Hz, H-3,4,5 Ph); 7.70 (d, 2H, ^3J =8.8 Hz, H-2,6 Ar); 9.40 (s, 1H, N=CH); 13.05 (br. s, 1H, NH). ^{13}C NMR (100 MHz, $CDCl_3$) δ: 18.7 (CH_3), 55.3 (OCH_3), 108.1, 113.9 (2C, Ar), 127.5 (2C, Ph), 128.1 (2C, Ph), 128.9, 130.0 (2C, Ar), 132.2, 138.1, 140.2, 145.4, 159.9, 161.3, 161.8, 162.0. Anal. Calcd for $C_{20}H_{18}N_2O_2$: C, 75.45; H, 5.70; N, 8.80. Found: C, 75.04; H, 6.06; N, 8.38.
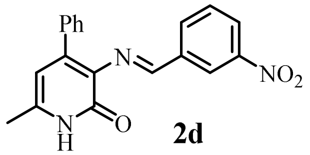<br>**2d**<br>Chemical Formula: $C_{19}H_{15}N_3O_3$<br>Molecular Weight: 333,35<br>Elemental Analysis: C, 68.46; H, 4.54; N, 12.61; O, 14.40	**6-Methyl-3-((3-nitrobenzylidene)amino)-4-phenylpyridin-2(1*H*)-one (2d)**<br>Yield: 260 mg (78%), orange crystals, m.p.: 290-291 °C.<br>IR (KBr, cm^{-1}): 1534, 1624, 1655, 2893<br>^1H NMR (400 MHz, DMSO-d6 + CF_3COOH) δ: 2.16 (s, 3H, CH_3); 5.98 (s, 1H, H-5); 7.38 (s, 5H, Ph); 7.70 (m, 1H, H-6 Ar); 8.17 (m, 1H, H-5 Ar); 8.36 (m, 1H, H-6 Ar); 8.55 (s, 1H, H-2 Ar); 9.99 (br. s, 1H, N=CH); 13.18 (br. s, 1H, NH). ^{13}C NMR (100 MHz, DMSO-d6) δ: 19.5 (CH_3), 108.2 (C-5), 116.9, 125.1, 129.0, 129.5 (2C Ph), 129.7, 130.4 (2C Ph), 130.9, 131.9, 136.1, 136.3, 138.8, 146.4, 148.2, 149.9, 192.3. Anal. Calcd for $C_{19}H_{15}N_3O_3$: C, 68.46; H, 4.54; N, 12.61. Found: C, 68.88; H, 4.93; N, 12.18.

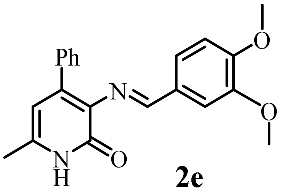<br>**2e**<br>Chemical Formula: $C_{21}H_{20}N_2O_3$<br>Molecular Weight: 348,40<br>Elemental Analysis: C, 72.40; H, 5.79; N, 8.04; O, 13.78	**3-((3,4-Dimethoxybenzylidene)amino)-6-methyl-4-phenylpyridin-2(1*H*)-one (2e)**<br>Yield: 294 mg (84%), yellow crystals, m.p.: 220-224 °C.<br>IR (KBr):,ν, cm^{-1}: 1271, 1583, 1605, 2831.<br>^{1}H NMR (500 MHz, DMSO-d_6) δ: 2.21 (s, 3H, CH_3); 3.79 (s, 3H, OCH_3); 3.84 (s, 3H, OCH_3); 6.13 (s, 1H, H-5); 6.53 (dd, 1H, ^3J = 8.7 Hz, ^5J = 2.1 Hz, H-5 Ar); 6.60 (d, 1H, ^5J = 2.2 Hz, H-2 Ar); 7.33-7.38 (m, 3H, H-3,4,5 Ph); 7.42-7.43 (m, 2H, H-2,6 Ph); 7.64 (d, 1H, ^3J = 8.8 Hz, H-6 Ar); 9.54 (s, 1H, N=CH); 11.81 (br. s, 1H, NH). ^{13}C NMR (125 MHz, DMSO-d_6) δ: 18.3 (CH_3), 55.4 (OCH_3), 55.6 (OCH_3), 98.0 (C-2, Ar), 106.2 (C-5), 106.5 (C-5 Ar), 118.4 (C-6 Ar), 127.4 (2C, Ph), 127.5, 127.7, 129.7 (2C, Ph), 132.1, 138.2, 140.2, 143.2, 155.5, 159.2, 160.4, 163.1. Anal. Calcd for $C_{21}H_{20}N_2O_3$: C, 72.40; H, 5.79; N, 8.04. Found: C, 72.05; H, 6.21; N, 8.39.
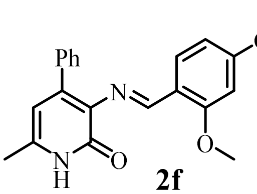<br>**2f**<br>Chemical Formula: $C_{21}H_{20}N_2O_3$<br>Molecular Weight: 348,40<br>Elemental Analysis: C, 72.40; H, 5.79; N, 8.04; O, 13.78	**3-((2,4-Dimethoxybenzylidene)amino)-6-methyl-4-phenylpyridin-2(1*H*)-one (2f)**<br>Yield: 296 mg (85%), yellow crystals, m.p.: 241-244 °C.<br>IR (KBr):,ν, cm^{-1}: 1230, 1262, 1621, 1635, 2838.<br>^{1}H NMR (500 MHz, DMSO-d_6) δ: 2.23 (s, 3H, CH_3); 3.68 (s, 3H, OCH_3); 3.78 (s, 3H, OCH_3); 6.19 (s, 1H, H-5); 6.79 (d, 1H, ^3J = 8.3 Hz, H-5 Ar); 7.22 (dd, 1H, ^3J = 8.2 Hz, ^5J = 1.6 Hz, H-6 Ar); 7.28 (d, 1H, ^5J = 1.5 Hz, H-3 Ar); 7.34 (t, 1H, ^3J = 7.2 Hz, H-4 Ph); 7.39 (t, 2H, ^3J = 7.3 Hz, H-3,5 Ph); 7.48 (d, 2H, ^3J = 7.1 Hz, H-2,6 Ph); 9.41 (s, 1H, N=CH); 11.91 (br. s, 1H, NH). ^{13}C NMR (125 MHz, DMSO-d_6) δ: 18.3 (CH_3), 55.1 (OCH_3), 55.6 (OCH_3), 106.6 (C-3 Ar), 109.0 (C-5), 111.3 (C-5 Ar) 123.0 (C-6 Ar), 127.4 (2C Ph), 127.8,

	129.9 (2C Ph), 130.6, 130.7, 138.1 140.1, 144.4, 148.9, 151.2, 159.3, 160.0. Anal. Calcd for $C_{21}H_{20}N_2O_3$: C, 72.40; H, 5.79; N, 8.04. Found: C, 72.08; H, 6.18; N, 8.41.
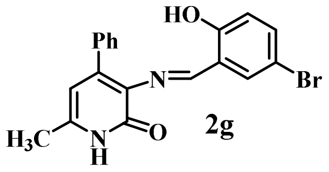<br>Chemical Formula: $C_{19}H_{15}BrN_2O_2$<br>Molecular Weight: 383,24<br>Elemental Analysis: C, 59.55; H, 3.95; Br, 20.85; N, 7.31; O, 8.35	**3-((5-Bromo-2-hydroxybenzylidene)amino)-6-methyl-4-phenylpyridin-2(1*H*)-one (2g)**<br>Yield: 362 mg (94%), yellow crystals, m.p.: 294-297 °C .<br>IR (KBr, cm^{-1}): 820, 1162, 1621, 1625, 2922.<br>^{1}H NMR (400 MHz, DMSO-d6) δ: 2.27 (s, 3H, CH_3); 6.14 (s, 1H, H-5); 6.69 (d, 1H, ^3J = 9.2 Hz, H-3 Ar); 7.36-7.44 (m, 6H, H-4 Ar, H-2,3,4,5,6 Ph); 7.63(s, 1H, H-6 Ar) 9.83 (s, 1H, N=CH); 12.03 (br. s, 1H, NH); 12.50 (s, 1H, OH). Anal. Calcd for $C_{19}H_{15}BrN_2O_2$: C, 59.55; H, 3.95; N, 7.31. Found: C, 59.98; H, 4.38; N, 7.70.
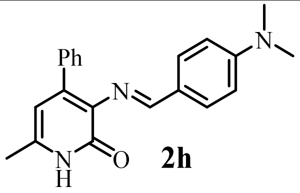<br>Chemical Formula: $C_{21}H_{21}N_3O$<br>Molecular Weight: 331,4190<br>Elemental Analysis: C, 76.11; H, 6.39; N, 12.68; O, 4.83	**3-((4-Dimethylamino)benzylidene)amino)-6-methyl-4-phenylpyridin-2(1*H*)-one** (**2h**). Yield: 310 mg (93%), yellow crystals, m.p.: 258-261°C (2-propanol). IR (KBr): ν, cm^{-1}: 3748, 2894, 1633, 1614. ^{1}H NMR (400 MHz, $CDCl_3$) δ: 2.36 (s, 3H, CH_3); 3.01 (s, 6H, $N(CH_3)_2$); 6.20 (s, 1H, H-5); 6.66 (d, 2H, *J*= 7.3, H-3',5' Ar); 7.33-7.36 (m, 3H, H-3,4,5 Ph); 7.51 (d, 2H, *J*= 7.3, H-2,6 Ph); 7.63 (d, 2H, *J*= 7.3, H-3',5' Ar); 9.20 (s, 1H, N=CH); 12.55 (br. s, 1H, NH). ^{13}C NMR (100 MHz, $CDCl_3$) δ ppm 18.8 (CH_3); 40.2 ($N(\underline{C}H_3)_2$) 108.5 (C-5); 111.4 (C-3',5' Ar); 125.7; 127.5 (C-3,5 Ph); 127.7 (C-4 Ph); 130.1 (C-2',6' Ar); 130.2 (C-2,6 Ph); 133.3 (C-1' Ar); 138.2; 139.1; 143.9; 152.2; 161.3 (C-2); 162.9 (N=$\underline{C}$H). Anal. Calcd for $C_{21}H_{21}N_3O$: C, 76.11; H, 6.39; N, 12.68. Found: C, 75.74; H, 6.81; N, 12.28.

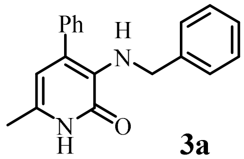<br>**3a**<br>Chemical Formula: $C_{19}H_{18}N_2O$<br>Molecular Weight: 290,3660<br>Elemental Analysis: C, 78.59; H, 6.25; N, 9.65; O, 5.51	**3-(Benzylamino)-6-methyl-4-phenylpyridin-2(1*H*)-one (3a)**<br>Yield: 235 mg (81%), yellow crystals, m.p.: 167-169 °C (2-propanol-hexane).<br>IR (KBr, cm^{-1}): 1628, 1831, 2925, 3274, 3334;<br>^{1}H NMR (400 MHz, $CDCl_3$) δ: 2.27 (s, 3H, CH_3); 3.73 (s, 2H, N-CH_2); 5.19 (br. s, 1H, N-H); 5.87 (s, 1H, H-5); 6.88 (m, 2H, H-2',6' Ar); 7.0 (m, 3H, H-3',4',5' Ar); 7.3 (m, 5H, Ph); 12.38 (s, 1 H, NHCO);<br>^{13}C NMR (100 MHz, $CDCl_3$) δ: 18.2; 49.9; 110.6; 126.5; 127.3; 127.6; 128.0; 128.2; 128.3; 132.2; 132.6; 132.9; 139.0; 139.9; 160.2.<br>Anal. Calcd for $C_{19}H_{18}N_2O$: C, 78.59; H, 6.25; N, 9.65; Found: C, 74.91; H, 6.05; N, 9.86.
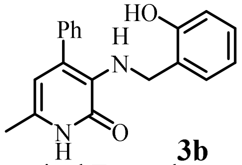<br>**3b**<br>Chemical Formula: $C_{19}H_{18}N_2O_2$<br>Molecular Weight: 306,3650<br>Elemental Analysis: C, 74.49; H, 5.92; N, 9.14; O, 10.4	**3-((2-Hydroxybenzyl)amino)-6-methyl-4-phenylpyridin-2(1*H*)-one (3b)**<br>Yield: 217 mg (71%), yellow crystals, m.p. 187-188 °C (2-propanol-hexane).<br>IR (KBr, cm^{-1}): 1542, 1632, 2920, 3367.<br>^{1}H NMR (400 MHz, $CDCl_3$) δ: 2.47 (s, 3H, CH_3); 3.62 (bt, 1H, *J=7.6* Hz, N-H); 4.24 (d, 2H, *J=6.9* Hz, N-CH_2); 6.10 (s, 1H, H-5); 6.74 (td, 1H, *J=7.3* Hz, *J=1.4* Hz, H-5' Ar); 6.91 (dd, 1H, $^{3}J=8.2$ Hz, $^{4}J=0.9$ Hz H-3' Ar), 6.99 (dd, 1H, *J=7.3* Hz, *J=1.4* Hz, H-6' Ar), 7.18 (td, 1H, *J=7.8* Hz, *J=1.4* Hz, H-4' Ar), 7.37-7.43 (m, 3H, H-3,4,5 Ph), 7.47 (d, 2H, *J=7.1* Hz, H-2,6 Ph), 10.05 (s, 1H, OH); 13.38 (s, 1H, NHCO).<br>^{13}C NMR (100 MHz, $CDCl_3$) δ: 18.6, 48.8, 109.5, 116.7, 118.5, 124.4, 127.8, 128.5, 129.2, 129.3, 131.8, 137.2, 137.4, 140.9 157.5, 162.7.<br>Anal. Calcd for $C_{19}H_{18}N_2O_2$: C, 74.49; H, 5.92; N, 9.14; Found: C, 74.81; H, 5.74; N, 9.26.

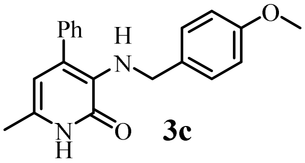<br>3c<br>Chemical Formula: $C_{20}H_{20}N_2O_2$<br>Molecular Weight: 320,39<br>Elemental Analysis: C, 74.98; H, 6.29; N, 8.74; O, 9.99	**3-((4-Methoxybenzyl)amino)-6-methyl-4-phenylpyridin-2(1*H*)-one (3c)**<br>Yield: 176 mg (55%), brown crystals, m.p.: 154-157°C °C (2-propanol-hexane).<br>IR (KBr, cm^{-1}): 1509, 1630, 1884, 2838, 3303.<br>^{1}H NMR (400 MHz, $CDCl_3$) δ: 2.27 (s, 3H, CH_3); 3,74 (s, 3H, OCH_3) 3.75 (s, 2H, N-CH_2); 5.00 (br. s, 1H, N-H); 5.91 (s, 1H, H-5); 6.74 (d, 2H, ^{3}J= 9.2 Hz, H-3,5 Ar); 6.97 (d, 2H, ^{3}J= 6.1 Hz, H-2,6 Ar); 7.32 (t, 1H, ^{3}J= 6.9 Hz, H-4 Ph); 7.38-7.41 (m, 2H, H-5,3 Ph); 7.45-7.47 (m, 2H, H-2,6 Ph); 12.77 (s, 1 H, NH). ^{13}C NMR (100 MHz, $CDCl_3$) δ: 18.3 (CH_3), 49.8 (N-CH_2), 55.2 (OCH_3), 109.4 (C-5), 113.5 (C-3,5 Ar), 127.5, 128.3 (4C Ph), 128.7 (C-2,6 Ar), 132.1, 132.3, 133.0, 139.3, 158.4, 161.6. Anal. Calcd for $C_{20}H_{20}N_2O_2$: C, 74.98; H, 6.29; N, 8.74; Found: C, 74.54; H, 6.66; N, 8.34.
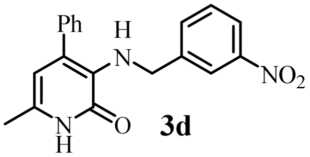<br>3d<br>Chemical Formula: $C_{19}H_{17}N_3O_3$<br>Molecular Weight: 335,36<br>Elemental Analysis: C, 68.05; H, 5.11; N, 12.53; O, 14.31	**6-Methyl-3-((3-nitrobenzyl)amino)-4-phenylpyridin-2(1*H*)-one (3d)**<br>Yield: 255 mg (76%), orange crystals, m.p.: 160-163°C (2-propanol).<br>IR (KBr, cm^{-1}): 1642,1844, 2923, 3303, 3359.<br>^{1}H NMR (400 MHz, $CDCl_3$) δ: 2.28 (s, 3H, CH_3); 3.96 (d, 2H, ^{3}J= 6.1 Hz, N-CH_2); 5.19 (br. s, 1H, N-H); 5.89 (s, 1H, H-5); 7.32-7.36 (m, 3H, H-3,4,5 Ph); 7.37-7.38 (m, 4H, H-2,6 Ph, H-5,6 Ar) 7.80 (s, 1H, H-2 Ar); 8.01 (d, 1H, ^{3}J= 6.1 Hz, H-4Ar) 12.74 (s, 1 H, NH). ^{13}C NMR (100 MHz, $CDCl_3$) δ: 18.3 (CH_3), 49.2 (N-CH_2), 109.4 (C-5), 121.9 (C-4 Ar), 122.4 (C-2 Ar), 127.9, 128.2 (2C Ph), 128.3 (2C Ph), 129.0, 131.9, 132.3, 132.9, 133.4, 142.4, 148.0, 161.5. Anal. Calcd for $C_{19}H_{17}N_3O_3$: C, 68.05; H, 5.11; N, 12.53; Found: C, 68.48; H, 5.52; N, 12.93.

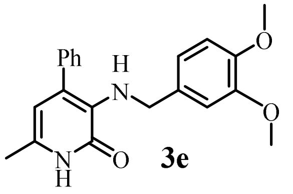<br>**3e**<br>Chemical Formula: $C_{21}H_{22}N_2O_3$<br>Molecular Weight: 350,42<br>Elemental Analysis: C, 71.98; H, 6.33; N, 7.99; O, 13.70	**3-((3,4-Dimethoxybenzyl)amino)-6-methyl-4-phenylpyridin-2(1*H*)-one (3e)**<br>Yield: 179 mg (51%), yellow crystals, m.p.: 132-133°C (2-propanol-hexane).<br>IR (KBr, cm^{-1}): 1518, 1629, 2931, 3341.2.<br>^{1}H NMR (400 MHz, $CDCl_3$) δ: 2.26 (s, 3H, CH_3); 3.75 (s, 2H, N-CH_2); 3,78 (s, 3H, OCH_3); 3,82 (s, 3H, OCH_3); 5.04 (br. s, 1H, N-H); 5.90 (s, 1H, H-5); 6.56 (s, 1H, H-2 Ar); 6.57 (d, 1H, ^3J = 7.4 Hz, H-5 Ar); 6.70 (d, 1H, ^3J = 7.3 Hz, H-6 Ar); 7.32 (t, 1H, ^3J = 7.3 Hz, H-4 Ph); 7.39 (t, 2H, ^3J = 7.9 Hz, H-3,5 Ph); 7.43 (m, 2H, H-2,6 Ph); 12.74 (br. s, 1 H, NH). ^{13}C NMR (100 MHz, $CDCl_3$) δ: 18.3 (CH_3), 50.0 (N-CH_2), 55.6 (OCH_3), 55.8 (OCH_3), 109.4 (C-5), 110.7 (C-2 Ar), 110.7 (C-5 Ar), 119.5 (C-6 Ar), 127.5, 128.3 (2C Ph), 128.3 (2C Ph), 131.6, 132.1, 132.8, 133.0, 139.2, 147.7, 148.6, 161.6. Anal. Calcd for $C_{21}H_{22}N_2O_3$: C, 71.98; H, 6.33; N, 7.99; Found: C, 72.39; H, 6.72; N, 8.36.
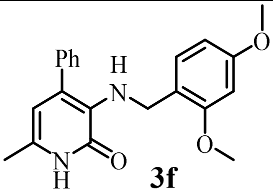<br>**3f**<br>Chemical Formula: $C_{21}H_{22}N_2O_3$<br>Molecular Weight: 350,42<br>Elemental Analysis: C, 71.98; H, 6.33; N, 7.99; O, 13.70	**3-((2,4-Dimethoxybenzyl)amino)-6-methyl-4-phenylpyridin-2(1*H*)-one (3f)**<br>Yield: 214 mg (61%), yellow crystals, m.p.: 181-184°C (2-propanol-hexane).<br>IR (KBr, cm^{-1}): 1644, 1865, 2938, 3304.<br>^{1}H NMR (400 MHz, $CDCl_3$) δ: 2.25 (s, 3H, CH_3); 3,64 (s, 3H, OCH_3); 3,75 (s, 3H, OCH_3) 3.84 (s, 2H, N-CH_2); 5.07 (br. s, 1H, N-H); 5.88 (s, 1H, H-5); 6.30 (d, 1H, ^3J = 7.7 Hz, H-5 Ar); 6.32 (s, 1H, H-3 Ar); 6.81 (d, 1H, ^3J = 7.6 Hz, H-6 Ar); 7.31 (t, 1H, ^3J = 6.9 Hz, H-4 Ph); 7.36-7.43 (m, 4H, H-2,3,5,6 Ph); 12.54 (s, 1 H, NH). ^{13}C NMR (100 MHz, $CDCl_3$) δ: 18.3 (CH_3), 45.0 (N-CH_2), 55.0 (4-OCH_3), 55.2 (2-OCH_3), 98.2 (C-3 Ar), 103.3 (C-5), 109.2 (C-5 Ar), 120.8, 127.3, 128.3 (4C Ph), 129.8, 131.7, 131.9, 133.3, 139.4, 154.5, 159.9,

	161.6. Anal. Calcd for $C_{21}H_{22}N_2O_3$: C, 71.98; H, 6.33; N, 7.99; Found: C, 71.54; H, 6.75; N, 8.42.
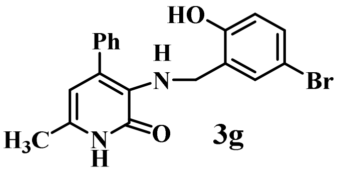<br>3g<br>Chemical Formula: $C_{19}H_{17}BrN_2O_2$<br>Molecular Weight: 385,26<br>Elemental Analysis: C, 59.23; H, 4.45; Br, 20.74; N, 7.27; O, 8.31	**3-((5-Bromo-2-hydroxybenzyl)amino)-6-methyl-4-phenylpyridin-2(1*H*)-one (3g)**<br>Yield: 285 mg (74%), yellow crystals, m.p.: 203-206 °C (2-propanol-hexane).<br>IR (KBr, cm^{-1}): 1643, 1842, 3279, 3370.<br>^{1}H NMR (400 MHz, DMSO-d6) δ: 2.09 (s, 3H, CH_3); 3.66 (d, *J* = 7.6 Hz, 2H, N-CH_2); 5.10 (bt, 1H, ^3J = 6.9 Hz, N-H); 5.81 (s, 1H, H-5); 6.62 (d, 1H, ^3J = 9.2 Hz, H-3 Ar); 6.83 (br. s, 1H, H-6 Ar); 7.11-7.14 (dd, 1H, $^3J=9.2$ Hz, $^4J=3.1$ Hz, H-4 Ar); 7.34 (td, 1H, $^3J=6.1$ Hz, $^4J=3.1$ Hz, H-4 Ph); 7.39-7.42 (m, 4H, H-3,2,5,6 Ph); 9.8 (s, 1H, OH); 11.6 (s, 1 H, NH). ^{13}C NMR (100 MHz, DMSO-d6) δ: 17.8 (CH_3), 44.5 (N-CH_2), 107.5 (C-5 Ar), 109.5 (C-5), 116.9 (C-4 Ar), 127.5 (C-3 Ar), 127.9 (2C Ph), 128.5 (2C Ph), 128.9, 129.1, 130.3 (C-6 Ar), 130.9, 132.0, 132.6, 139.0, 154.7, 160.1. Anal. Calcd for $C_{19}H_{17}BrN_2O_2$: C, 59.23; H, 4.45; N, 7.27; Found: C, 59.63; H, 4.84; N, 7.70.
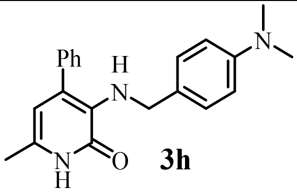<br>3h<br>Chemical Formula: $C_{21}H_{23}N_3O$<br>Molecular Weight: 333,4350<br>Elemental Analysis: C, 75.65; H, 6.95; N, 12.60; O, 4.80	**3-((4-Dimethylaminobenzyl)amino)-6-methyl-4-phenylpyridin-2(1*H*)-one (3h).** Yield: 250 mg (75%), yellow crystals, m.p.: 165-168°C (2-propanol-hexane). IR (KBr, cm^{-1}): 2922, 1643, 1518. ^{1}H NMR (400 MHz, $CDCl_3$) δ: 2.27 (s, 3H, CH_3); 2.89 (s, 6H, $N(CH_3)_2$); 3.70 (s, 2H, N-C$\underline{H}_2$); 4.97 (br. s, 1H, N-H); 5.93 (s, 1H, H-5); 6.60 (d, 2H, *J*= 9.2, H-3',5' Ar); 6.95 (d, 2H, *J*= 8.5 , H-3',5' Ar); 7.32 (t, 1H, *J*= 7.3, H-4 Ph); 7.41 (t, *J*= 7.3, 2H, H-3,5 Ph); 7.49 (d, 2H, *J*= 7.3, H-2,6 Ph); 12.83 (br. s, 1H, NH). ^{13}C NMR (100 MHz, $CDCl_3$) δ: 18.3 (CH_3); 40.7 (N($\underline{C}H_3)_2$); 50.0 (CH_2-Ar); 109.4 (C-5); 112.5 (C-3',5' Ar); 127.4 (C-4 Ph); 128.2; 128.2 (C-2',6' Ar); 128.3 (C-2,6 Ph);

	128.5 (C-3,5 Ph); 131.0; 131.9; 133.4; 139.5; 149.6 (C-4' Ar); 161.6 (C-2). Anal. Calcd for $C_{21}H_{23}N_3O$: C, 75.65; H, 6.95; N, 12.60; Found: C, 75.22; H, 5.65; N, 9.94.

Supporting information

NMR Spectral Data

Figure S1. 3-(Benzylideneamino)-6-methyl-4-phenylpyridin-2(1*H*)-one (2a)

^{1}H NMR (400 MHz, $CDCl_3$).

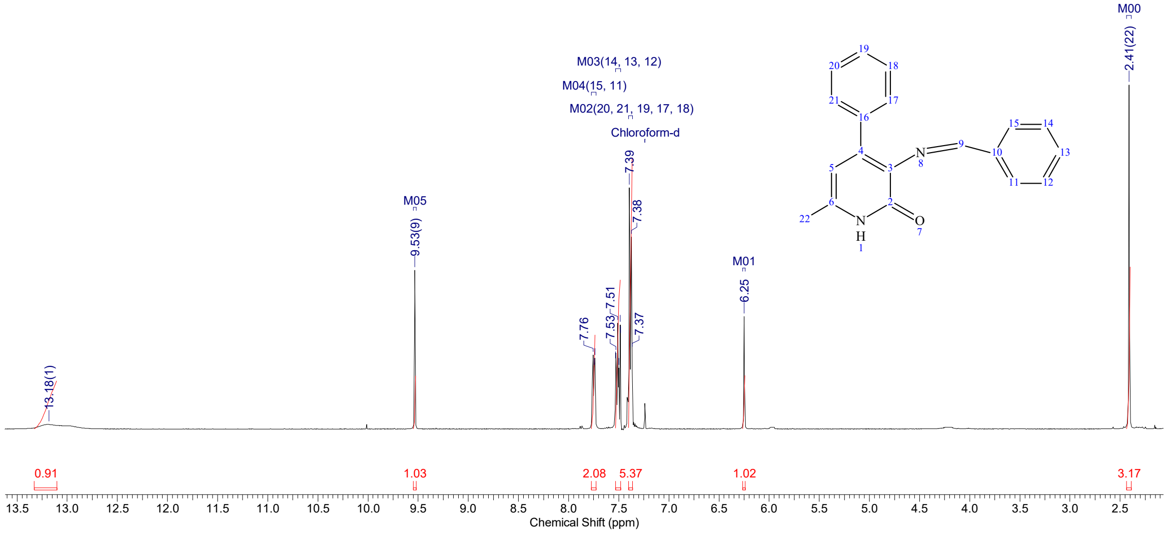

Figure S2. 3-(Benzylideneamino)-6-methyl-4-phenylpyridin-2(1*H*)-one (2a)

^{13}C NMR (100 MHz).

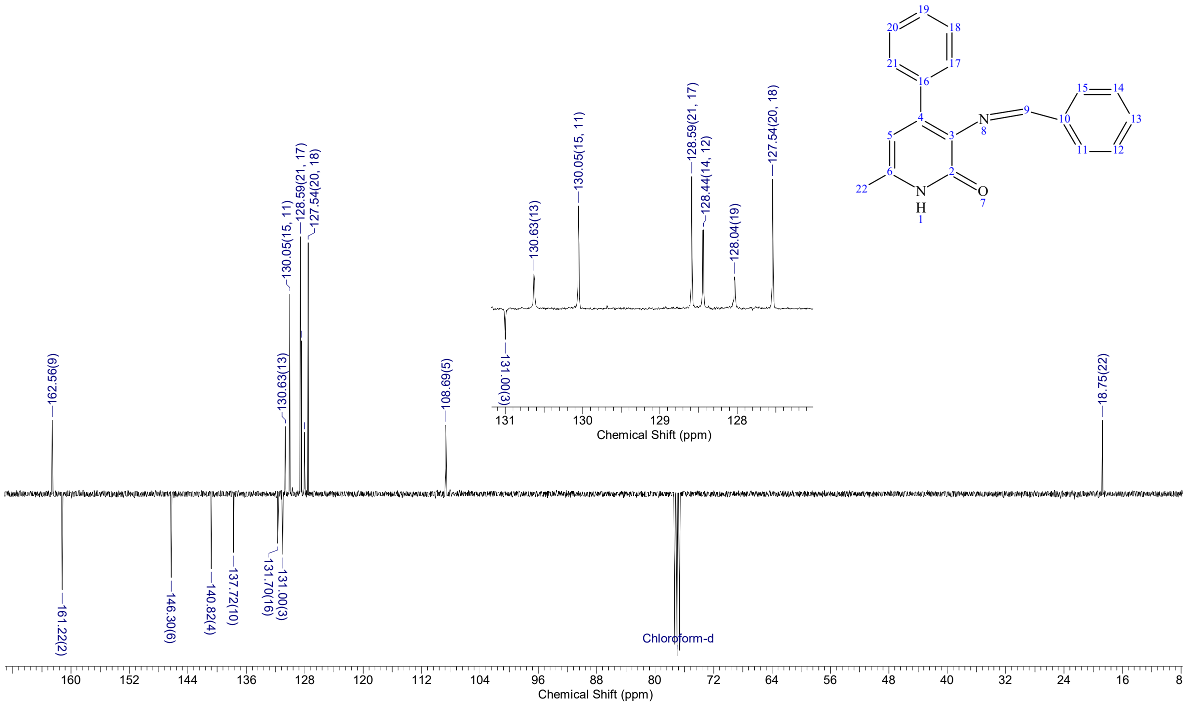

Figure S3. 3-((2-Hydroxybenzylidene)amino)-6-methyl-4-phenylpyridin-2(1*H*)-one (2b). ^{1}H NMR (400 MHz, DMSO-d6).

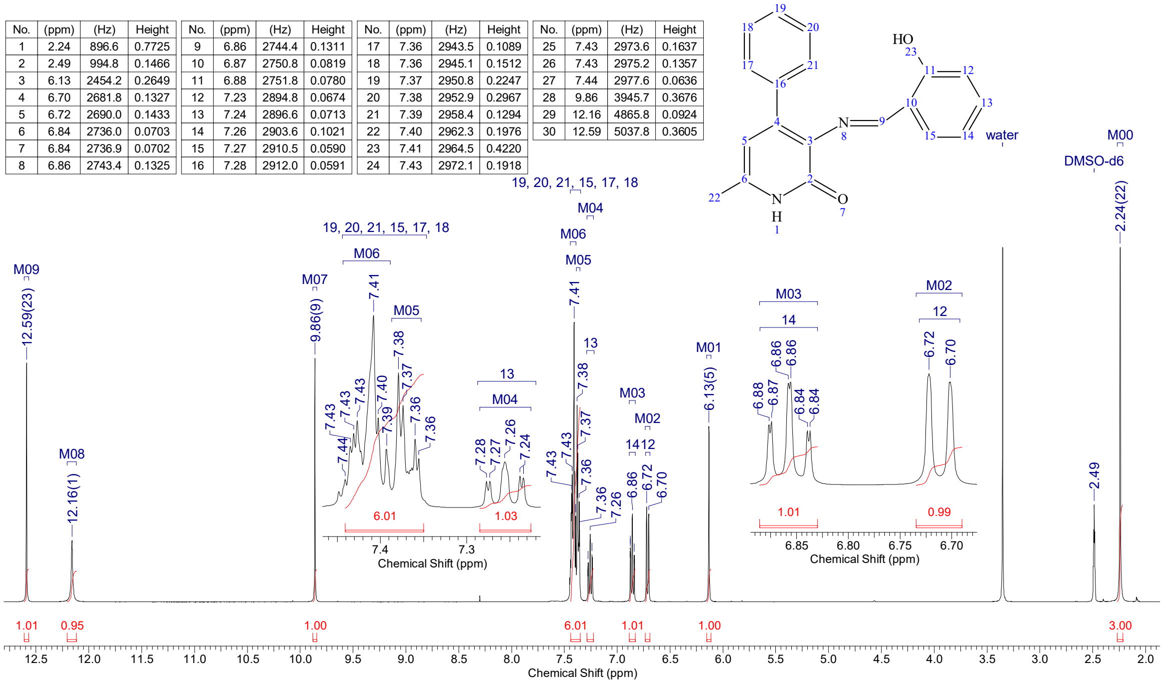

No.	(ppm)	(Hz)	Height
1	2.24	896.6	0.7725
2	2.49	994.8	0.1466
3	6.13	2454.2	0.2649
4	6.70	2681.8	0.1327
5	6.72	2690.0	0.1433
6	6.84	2736.0	0.0703
7	6.84	2736.9	0.0702
8	6.86	2743.4	0.1325

No.	(ppm)	(Hz)	Height
9	6.86	2744.4	0.1311
10	6.87	2750.8	0.0819
11	6.88	2751.8	0.0780
12	7.23	2894.8	0.0674
13	7.24	2896.6	0.0713
14	7.26	2903.6	0.1021
15	7.27	2910.5	0.0590
16	7.28	2912.0	0.0591

No.	(ppm)	(Hz)	Height
17	7.36	2943.5	0.1089
18	7.36	2945.1	0.1512
19	7.37	2950.8	0.2247
20	7.38	2952.9	0.2967
21	7.39	2958.4	0.1294
22	7.40	2962.3	0.1976
23	7.41	2964.5	0.4220
24	7.43	2972.1	0.1918

No.	(ppm)	(Hz)	Height
25	7.43	2973.6	0.1637
26	7.43	2975.2	0.1357
27	7.44	2977.6	0.0636
28	9.86	3945.7	0.3676
29	12.16	4865.8	0.0924
30	12.59	5037.8	0.3605

Figure S4. 3-((2-Hydroxybenzylidene)amino)-6-methyl-4-phenylpyridin-2(1*H*)-one (2b). ^{13}C NMR (100 MHz, DMSO-d6).

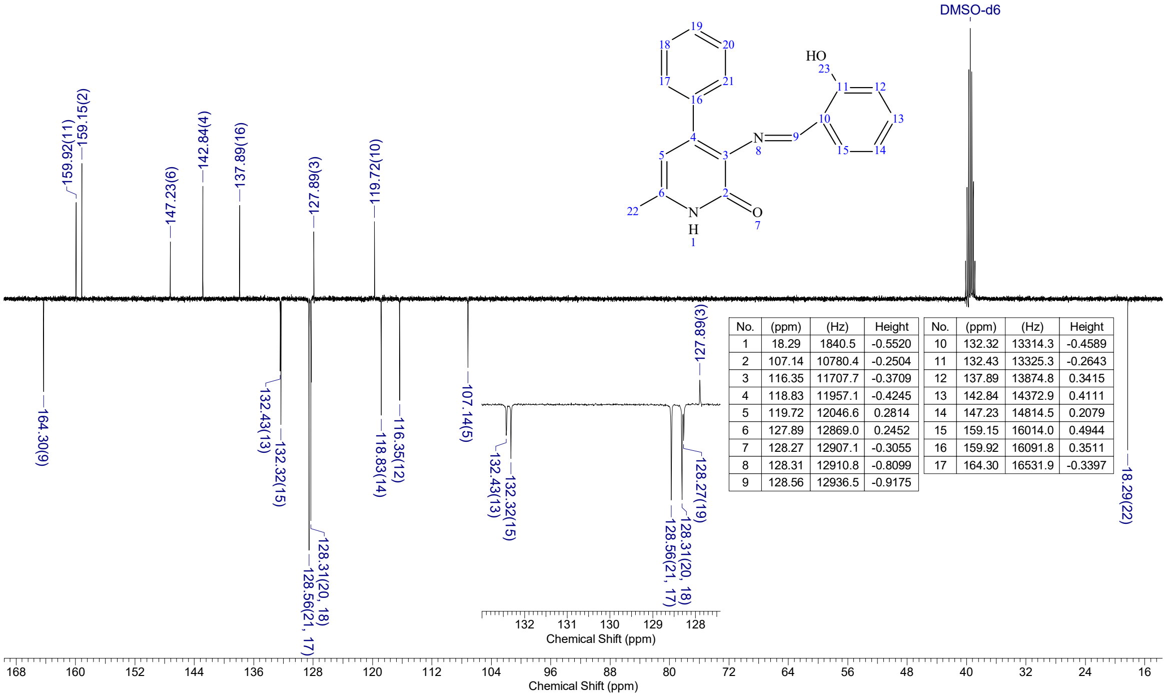

No.	(ppm)	(Hz)	Height
1	18.29	1840.5	-0.5520
2	107.14	10780.4	-0.2504
3	116.35	11707.7	-0.3709
4	118.83	11957.1	-0.4245
5	119.72	12046.6	0.2814
6	127.89	12869.0	0.2452
7	128.27	12907.1	-0.3055
8	128.31	12910.8	-0.8099
9	128.56	12936.5	-0.9175

No.	(ppm)	(Hz)	Height
10	132.32	13314.3	-0.4589
11	132.43	13325.3	-0.2643
12	137.89	13874.8	0.3415
13	142.84	14372.9	0.4111
14	147.23	14814.5	0.2079
15	159.15	16014.0	0.4944
16	159.92	16091.8	0.3511
17	164.30	16531.9	-0.3397

Figure S5. 3-((4-Methoxybenzylidene)amino)-6-methyl-4-phenylpyridin-2(1*H*)-one (2c)

^{1}H NMR (400 MHz, $CDCl_3$).

No.	(ppm)	(Hz)	Height	No.	(ppm)	(Hz)	Height
1	2.40	959.0	0.1433	10	7.48	2995.1	0.0405
2	3.83	1530.8	0.2944	11	7.50	3003.0	0.0489
3	6.23	2493.3	0.0482	12	7.51	3004.3	0.0524
4	6.23	2494.1	0.0490	13	7.52	3011.0	0.0360
5	6.89	2758.0	0.0549	14	7.53	3012.5	0.0301
6	6.91	2766.8	0.0582	15	7.69	3075.5	0.0581
7	7.37	2947.4	0.0484	16	7.71	3084.3	0.0553
8	7.38	2954.8	0.0521	17	9.40	3760.8	0.0684
9	7.48	2993.8	0.0330	18	13.05	5220.6	0.0022

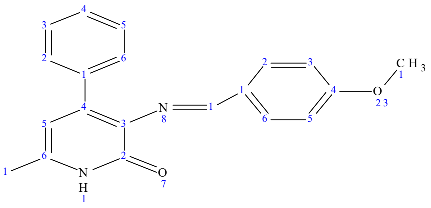

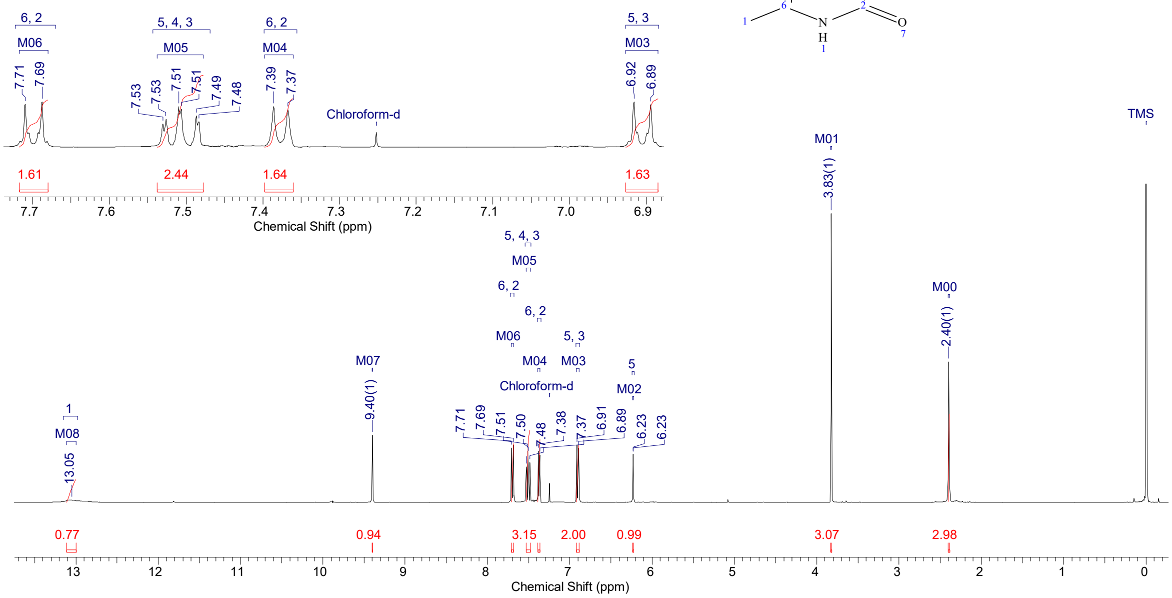

Figure S6. 3-((4-Methoxybenzylidene)amino)-6-methyl-4-phenylpyridin-2(1*H*)-one (2c)

^{13}C NMR (100 MHz).

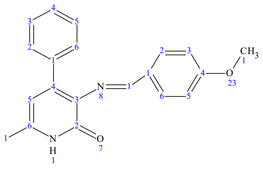

No.	(ppm)	(Hz)	Height	No.	(ppm)	(Hz)	Height
1	18.75	1886.3	0.4458	9	132.21	13303.6	-0.2831
2	55.29	5563.2	0.5438	10	138.10	13895.7	-0.2565
3	108.07	10873.9	0.2380	11	140.17	14104.0	-0.3287
4	113.88	11459.3	1.0000	12	145.35	14625.6	-0.1912
5	127.52	12831.2	0.6630	13	159.89	16088.5	-0.1427
6	128.06	12885.5	0.7701	14	161.27	16227.8	-0.2577
7	128.92	12972.1	0.3799	15	161.78	16279.2	-0.4254
8	130.22	13102.6	0.9056	16	162.04	16304.9	0.3678

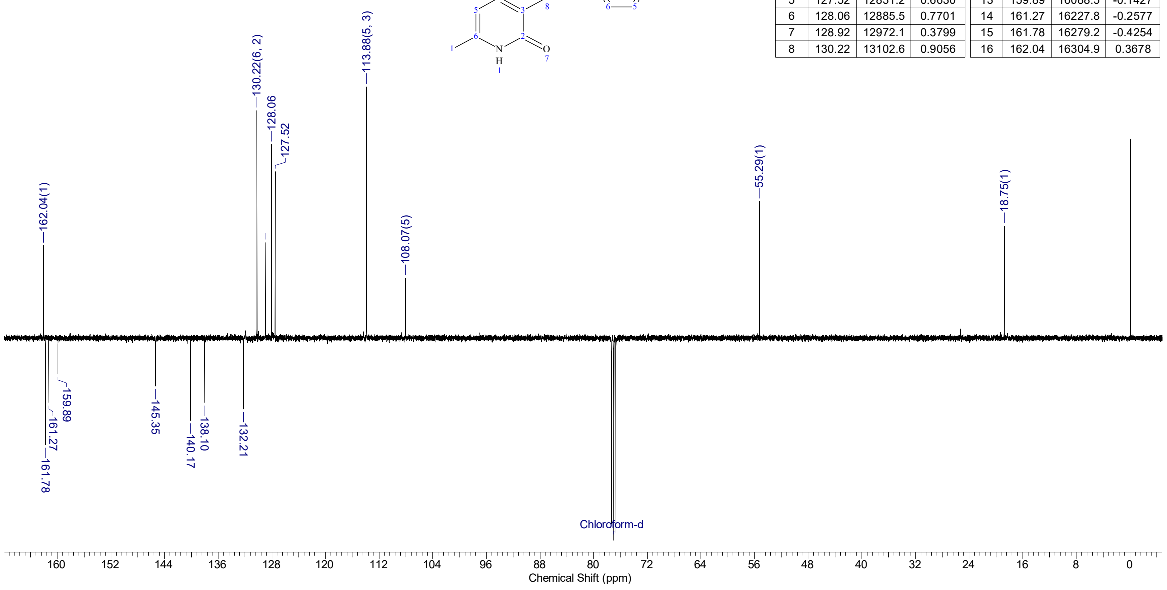

Figure S7. 6-methyl-3-((3-nitrobenzylidene)amino)-4-phenylpyridin-2(1*H*)-one (2d)

^{1}H NMR (400 MHz, DMSO-d6 + CF_3COOH).

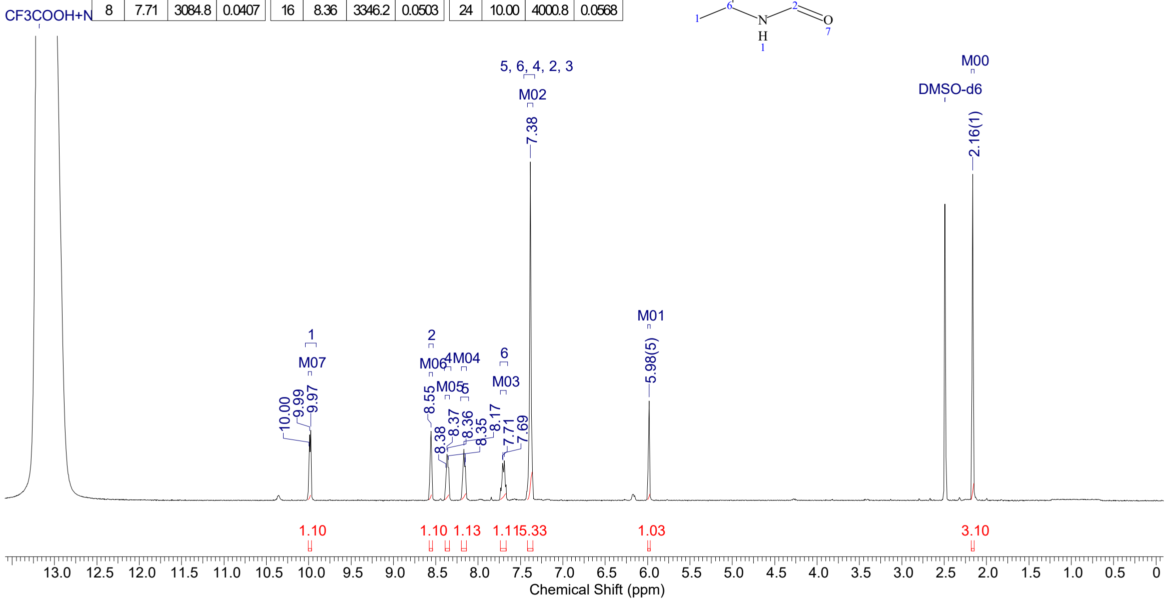

No.	(ppm)	(Hz)	Height	No.	(ppm)	(Hz)	Height	No.	(ppm)	(Hz)	Height
1	2.16	866.0	0.3730	9	7.71	3086.8	0.0414	17	8.36	3347.3	0.0515
2	5.98	2393.3	0.1131	10	8.15	3261.5	0.0385	18	8.37	3348.5	0.0522
3	7.38	2953.3	0.3862	11	8.15	3262.8	0.0347	19	8.38	3353.2	0.0322
4	7.38	2954.5	0.3869	12	8.17	3267.9	0.0546	20	8.55	3423.3	0.0785
5	7.69	3077.6	0.0448	13	8.17	3269.1	0.0576	21	9.97	3991.6	0.0791
6	7.69	3078.5	0.0446	14	8.35	3340.3	0.0407	22	9.98	3992.5	0.0749
7	7.70	3083.0	0.0377	15	8.35	3341.5	0.0409	23	9.99	3997.0	0.0743
8	7.71	3084.8	0.0407	16	8.36	3346.2	0.0503	24	10.00	4000.8	0.0568

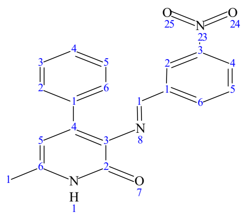

Figure S8. 6-methyl-3-((3-nitrobenzylidene)amino)-4-phenylpyridin-2(1*H*)-one (2d)

^{13}C NMR (100 MHzDMSO-d6).

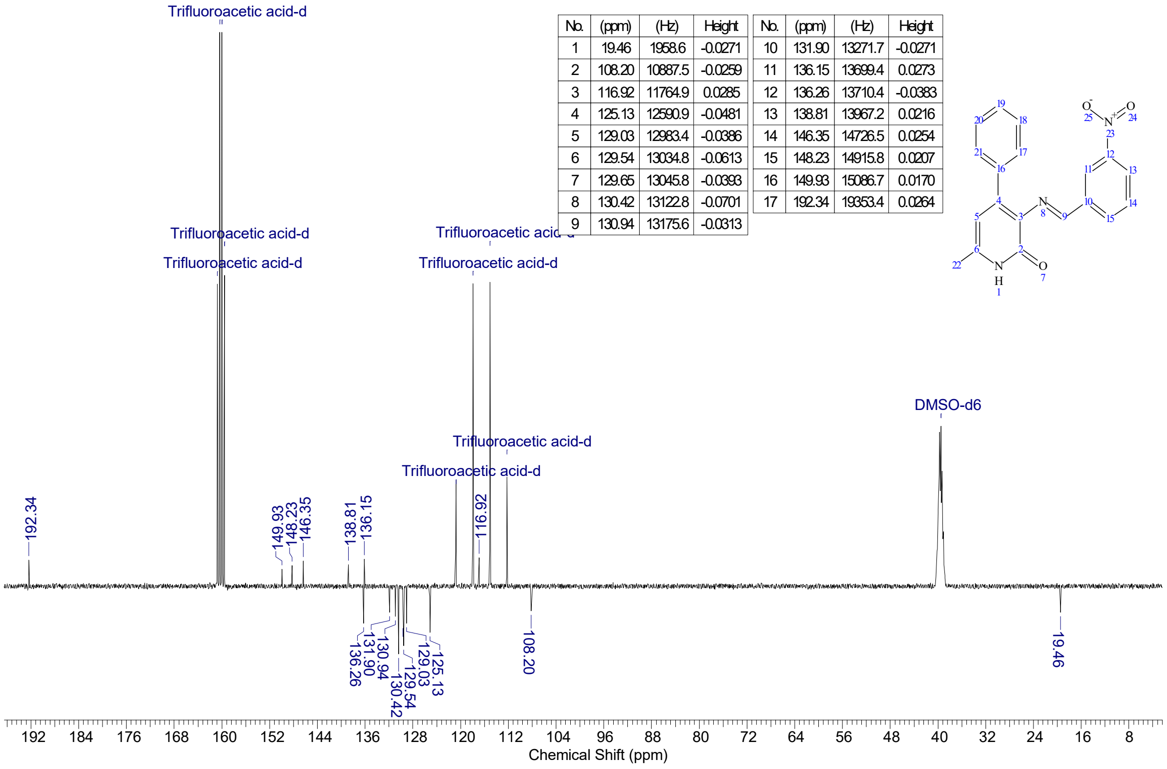

No.	(ppm)	(Hz)	Height
1	19.46	1958.6	-0.0271
2	108.20	10887.5	-0.0259
3	116.92	11764.9	0.0285
4	125.13	12590.9	-0.0481
5	129.03	12983.4	-0.0386
6	129.54	13034.8	-0.0613
7	129.65	13045.8	-0.0393
8	130.42	13122.8	-0.0701
9	130.94	13175.6	-0.0313

No.	(ppm)	(Hz)	Height
10	131.90	13271.7	-0.0271
11	136.15	13699.4	0.0273
12	136.26	13710.4	-0.0383
13	138.81	13967.2	0.0216
14	146.35	14726.5	0.0254
15	148.23	14915.8	0.0207
16	149.93	15086.7	0.0170
17	192.34	19353.4	0.0264

Figure S9. 3-((3,4-Dimethoxybenzylidene)amino)-6-methyl-4-phenylpyridin-2(1*H*)-one (2e) ^{1}H NMR (500 MHz, DMSO-d6).

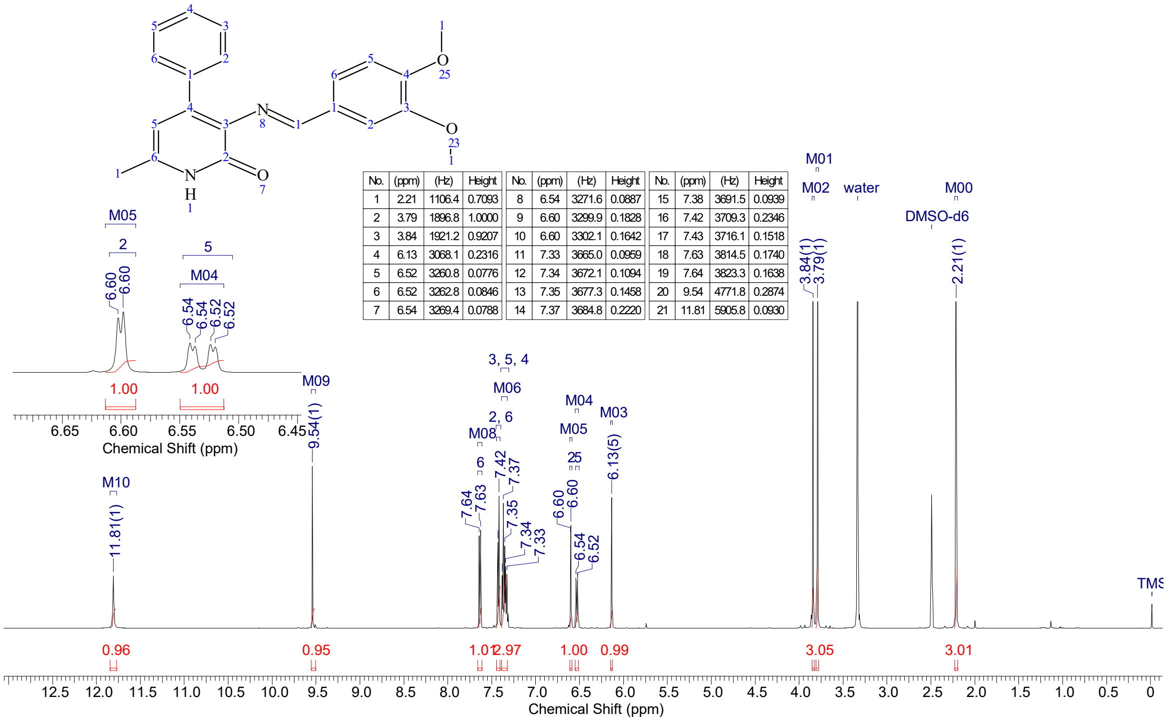

No.	(ppm)	(Hz)	Height
1	2.21	1106.4	0.7093
2	3.79	1896.8	1.0000
3	3.84	1921.2	0.9207
4	6.13	3068.1	0.2316
5	6.52	3260.8	0.0776
6	6.52	3262.8	0.0846
7	6.54	3269.4	0.0788

No.	(ppm)	(Hz)	Height
8	6.54	3271.6	0.0887
9	6.60	3299.9	0.1828
10	6.60	3302.1	0.1642
11	7.33	3665.0	0.0959
12	7.34	3672.1	0.1094
13	7.35	3677.3	0.1458
14	7.37	3684.8	0.2220

No.	(ppm)	(Hz)	Height
15	7.38	3691.5	0.0939
16	7.42	3709.3	0.2346
17	7.43	3716.1	0.1518
18	7.63	3814.5	0.1740
19	7.64	3823.3	0.1638
20	9.54	4771.8	0.2874
21	11.81	5905.8	0.0930

Figure S10. 3-((3,4-Dimethoxybenzylidene)amino)-6-methyl-4-phenylpyridin-2(1*H*)-one (2e) ^{13}C NMR (125 MHz).

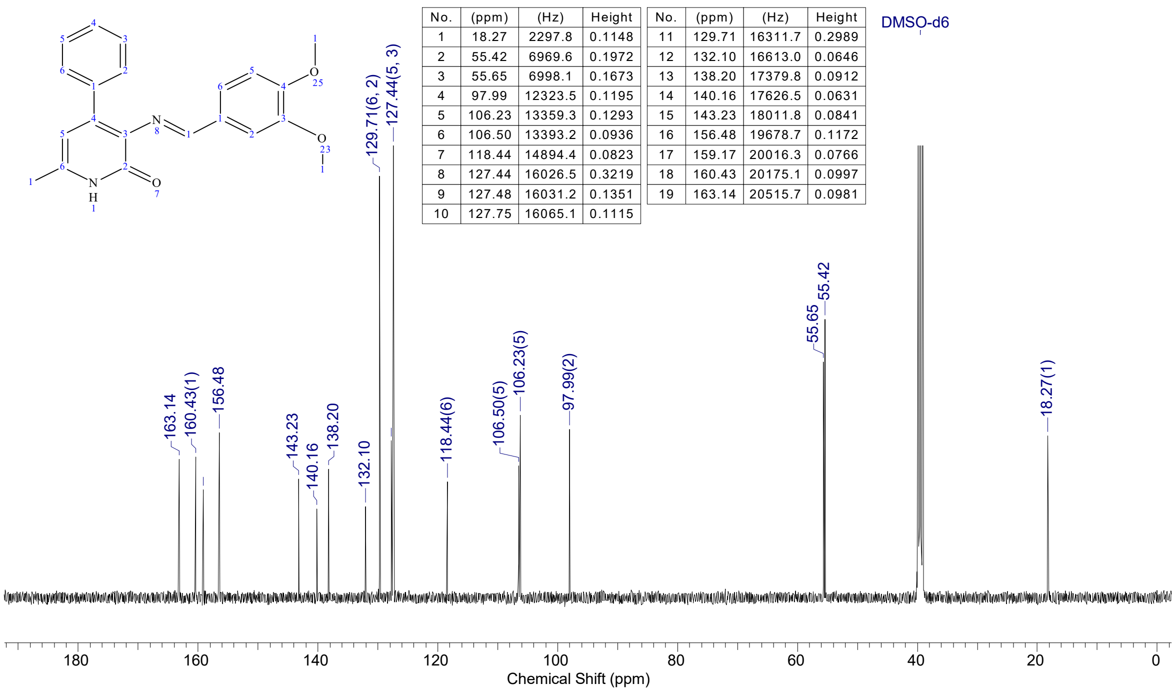

No.	(ppm)	(Hz)	Height
1	18.27	2297.8	0.1148
2	55.42	6969.6	0.1972
3	55.65	6998.1	0.1673
4	97.99	12323.5	0.1195
5	106.23	13359.3	0.1293
6	106.50	13393.2	0.0936
7	118.44	14894.4	0.0823
8	127.44	16026.5	0.3219
9	127.48	16031.2	0.1351
10	127.75	16065.1	0.1115
11	129.71	16311.7	0.2989
12	132.10	16613.0	0.0646
13	138.20	17379.8	0.0912
14	140.16	17626.5	0.0631
15	143.23	18011.8	0.0841
16	156.48	19678.7	0.1172
17	159.17	20016.3	0.0766
18	160.43	20175.1	0.0997
19	163.14	20515.7	0.0981

Figure S11. 3-((2,4-Dimethoxybenzylidene)amino)-6-methyl-4-phenylpyridin-2(1*H*)-one (2f) ^{1}H NMR (500 MHz, DMSO-d6).

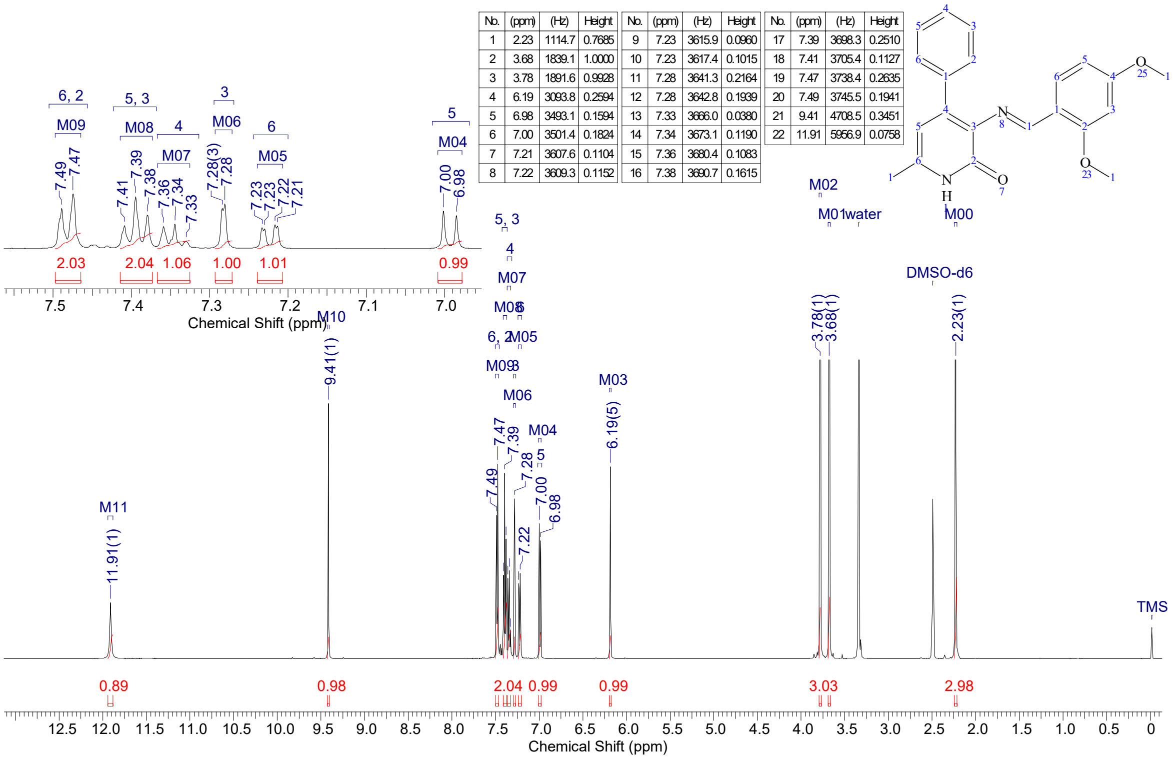

No.	(ppm)	(Hz)	Height	No.	(ppm)	(Hz)	Height	No.	(ppm)	(Hz)	Height
1	2.23	1114.7	0.7685	9	7.23	3615.9	0.0960	17	7.39	3698.3	0.2510
2	3.68	1839.1	1.0000	10	7.23	3617.4	0.1015	18	7.41	3705.4	0.1127
3	3.78	1891.6	0.9928	11	7.28	3641.3	0.2164	19	7.47	3738.4	0.2635
4	6.19	3093.8	0.2594	12	7.28	3642.8	0.1939	20	7.49	3745.5	0.1941
5	6.98	3493.1	0.1594	13	7.33	3666.0	0.0380	21	9.41	4708.5	0.3451
6	7.00	3501.4	0.1824	14	7.34	3673.1	0.1190	22	11.91	5956.9	0.0758
7	7.21	3607.6	0.1104	15	7.36	3680.4	0.1083				
8	7.22	3609.3	0.1152	16	7.38	3690.7	0.1615				

Figure S12. 3-((2,4-Dimethoxybenzylidene)amino)-6-methyl-4-phenylpyridin-2(1*H*)-one (2f) ^{13}C NMR (125 MHz).

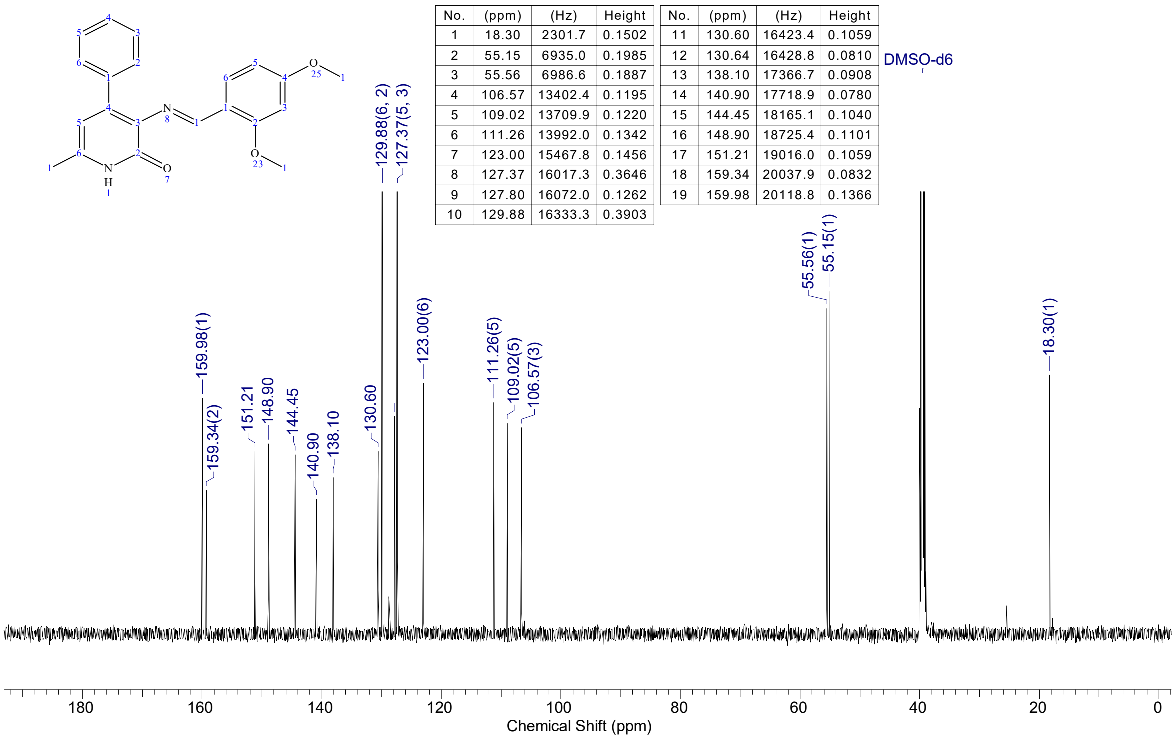

No.	(ppm)	(Hz)	Height	No.	(ppm)	(Hz)	Height
1	18.30	2301.7	0.1502	11	130.60	16423.4	0.1059
2	55.15	6935.0	0.1985	12	130.64	16428.8	0.0810
3	55.56	6986.6	0.1887	13	138.10	17366.7	0.0908
4	106.57	13402.4	0.1195	14	140.90	17718.9	0.0780
5	109.02	13709.9	0.1220	15	144.45	18165.1	0.1040
6	111.26	13992.0	0.1342	16	148.90	18725.4	0.1101
7	123.00	15467.8	0.1456	17	151.21	19016.0	0.1059
8	127.37	16017.3	0.3646	18	159.34	20037.9	0.0832
9	127.80	16072.0	0.1262	19	159.98	20118.8	0.1366
10	129.88	16333.3	0.3903				

Figure S13. 3-((5-Bromo-2-hydroxybenzylidene)amino)-6-methyl-4-phenylpyridin-2(1*H*)-one (2g)

^{1}H NMR (400 MHz, DMSO-d6).

No.	(ppm)	(Hz)	Height
1	2.27	906.9	0.4456
2	2.49	995.5	1.0000
3	6.14	2452.7	0.1371
4	6.69	2673.9	0.0796
5	6.71	2683.1	0.0943
6	7.36	2944.0	0.1566
7	7.38	2951.6	0.1980
8	7.42	2965.4	0.2313
9	7.44	2973.0	0.1212
10	7.63	3052.3	0.1165
11	9.83	3928.2	0.1601
12	12.03	4808.6	0.0275
13	12.50	4996.3	0.1209

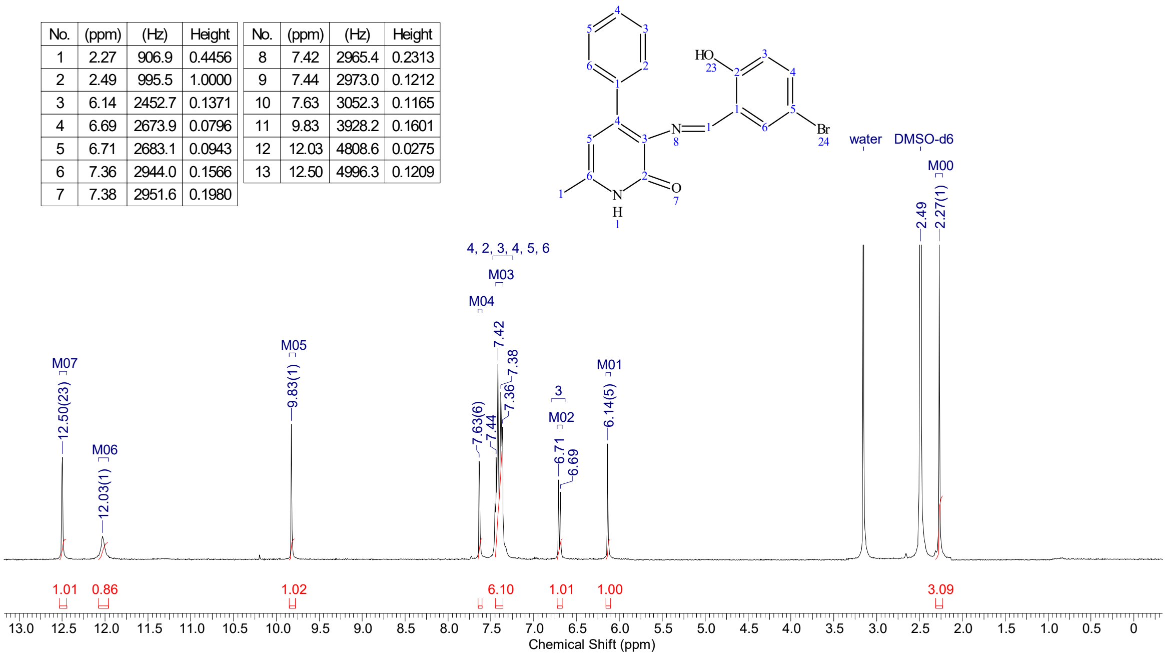

Figure S14. 3-((4-Dimethylamino)benzylidene)amino)-6-methyl-4-phenylpyridin-2(1*H*)-one (2h).

^{1}H NMR (400 MHz, DMSO-d6).

No.	(ppm)	(Hz)	Height
1	2.36	942.9	0.4398
2	3.01	1202.9	1.0000
3	6.20	2477.9	0.1535
4	6.66	2664.0	0.1468
5	6.68	2671.4	0.1607
6	7.33	2930.2	0.0969
7	7.34	2935.7	0.1291

No.	(ppm)	(Hz)	Height
8	7.36	2943.0	0.1555
9	7.51	3001.6	0.1633
10	7.53	3008.9	0.1493
11	7.63	3049.2	0.1539
12	7.65	3056.5	0.1527
13	9.20	3678.5	0.1546
14	12.55	5015.7	0.0368

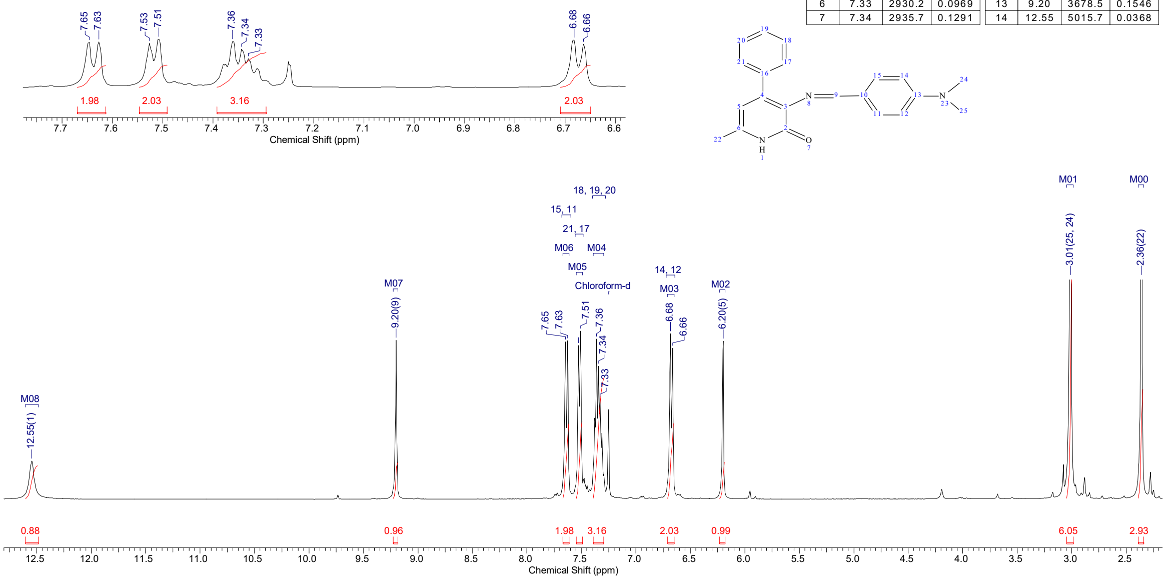

Figure S15. 3-((4-Dimethylamino)benzylidene)amino)-6-methyl-4-phenylpyridin-2(1*H*)-one (2h)

^{13}C NMR (100 MHz).

No.	(ppm)	(Hz)	Height	No.	(ppm)	(Hz)	Height
1	18.79	1889.0	0.0908	9	130.23	13091.6	0.1892
2	40.18	4038.9	0.1814	10	133.31	13401.2	0.0471
3	108.52	10909.1	0.0741	11	138.19	13891.9	0.0540
4	111.44	11202.4	0.1935	12	139.14	13986.8	0.0540
5	125.67	12633.4	0.0606	13	143.93	14468.9	0.0491
6	127.50	12817.4	0.2027	14	152.18	15298.0	0.0588
7	127.75	12842.4	0.0829	15	161.28	16212.3	0.0559
8	130.08	13076.2	0.2080	16	162.92	16377.2	0.0914

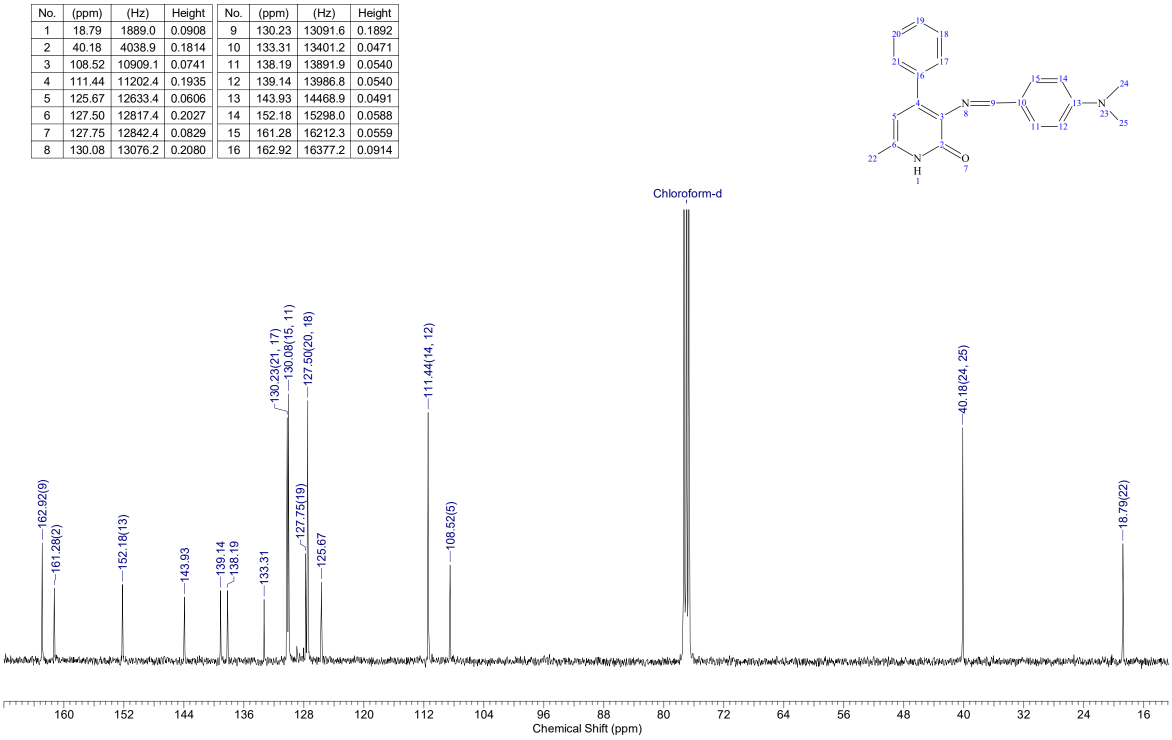

Figure S16. 3-(Benzylamino)-6-methyl-4-phenylpyridin-2(1*H*)-one (3a)

^{1}H NMR (400 MHz, $CDCl_3$).

No.	(ppm)	(Hz)	Height	No.	(ppm)	(Hz)	Height	No.	(ppm)	(Hz)	Height
1	2.27	907.0	1.0000	9	7.15	2858.3	0.0397	17	7.32	2927.2	0.1827
2	3.81	1521.6	0.2615	10	7.17	2865.6	0.1648	18	7.34	2934.0	0.1794
3	3.82	1527.7	0.2785	11	7.18	2872.3	0.2235	19	7.38	2948.6	0.1959
4	5.07	2026.4	0.1051	12	7.20	2877.2	0.3465	20	7.39	2955.9	0.4495
5	5.08	2031.9	0.0556	13	7.21	2883.9	0.3364	21	7.41	2963.3	0.2449
6	5.91	2363.9	0.3116	14	7.23	2890.6	0.1077	22	7.44	2973.6	0.4307
7	7.04	2816.2	0.2511	15	7.25	2899.2	0.2081	23	7.45	2980.3	0.2246
8	7.06	2822.9	0.2953	16	7.30	2919.9	0.0605	24	12.63	5048.8	0.0116

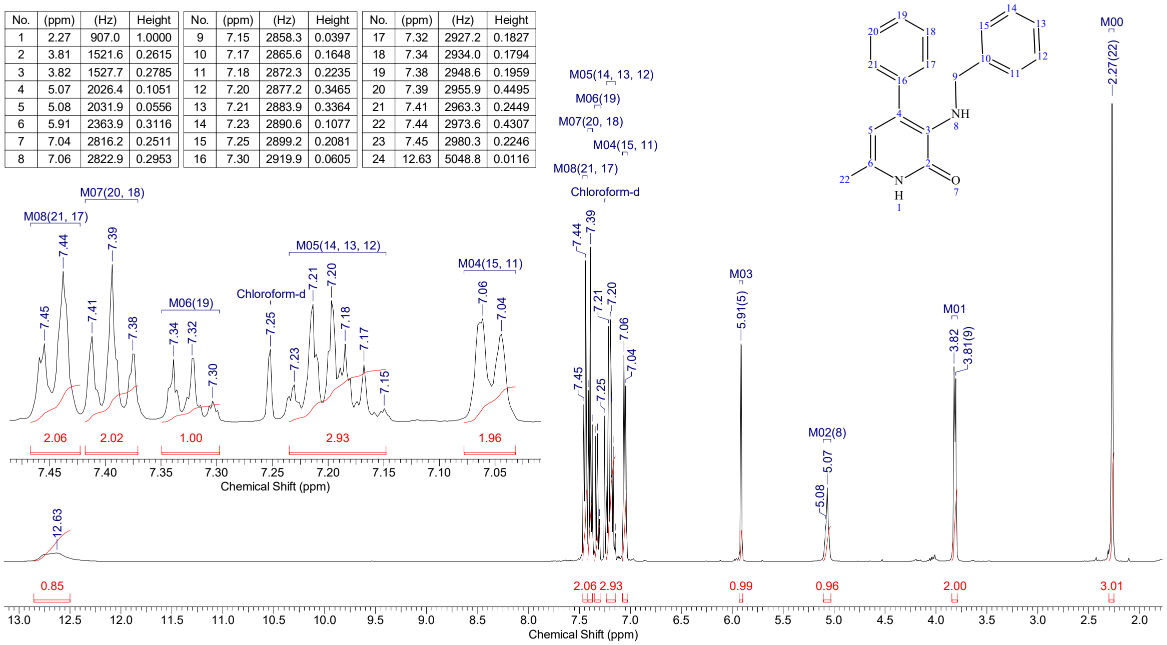

Figure S17. 3-(Benzylamino)-6-methyl-4-phenylpyridin-2(1*H*)-one (3a)

^{13}C NMR (100 MHz).

No.	Atom	Exp. Shift (ppm)
1	1	18.22
2	1	49.91
3	5	110.59
4	4	126.48
5	6	127.27
6	2	127.27
7	4	127.62

No.	Atom	Exp. Shift (ppm)
8	5	128.04
9	3	128.04
10	5	128.25
11	3	128.25
12	6	128.34
13	2	128.34
14	3	132.18

No.	Atom	Exp. Shift (ppm)
15	4	132.61
16	1	132.92
17	1	139.05
18	6	139.95
19	2	160.25

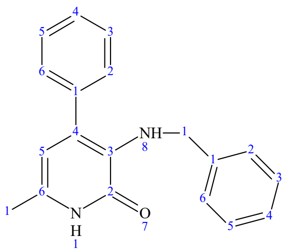

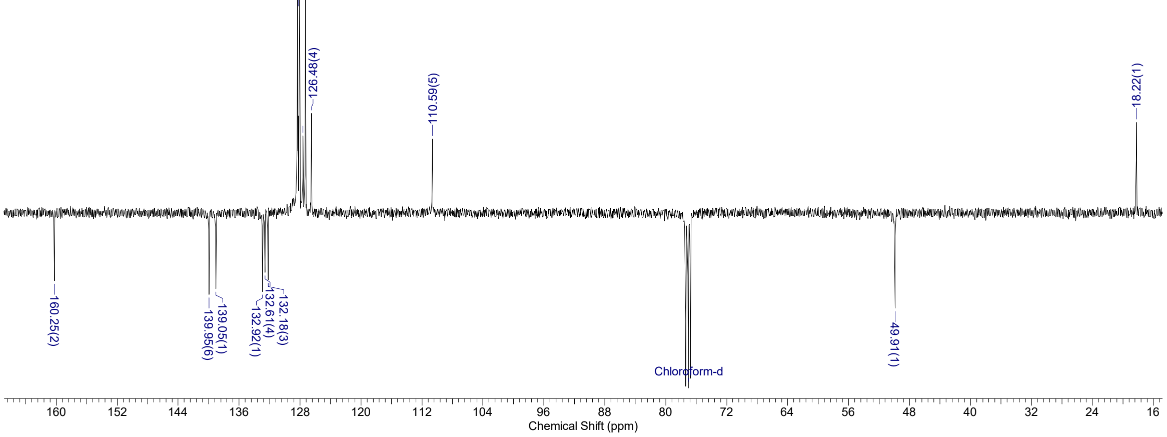

Figure S18. 3-((2-Hydroxybenzyl)amino)-6-methyl-4-phenylpyridin-2(1*H*)-one (3b) ^{1}H NMR (400 MHz, $CDCl_3$).

No.	(ppm)	(Hz)	Height
1	2.47	986.4	1.0000
2	3.60	1439.8	0.0307
3	3.62	1447.1	0.0641
4	3.64	1454.9	0.0356
5	4.23	1690.7	0.1730
6	4.25	1697.6	0.1638
7	6.10	2439.9	0.2727
8	6.72	2684.9	0.0769
9	6.72	2686.3	0.0782
10	6.73	2692.3	0.1619

No.	(ppm)	(Hz)	Height
11	6.74	2693.6	0.1682
12	6.75	2699.6	0.0917
13	6.76	2701.0	0.0964
14	6.90	2756.8	0.1527
15	6.90	2757.8	0.1542
16	6.92	2765.1	0.1845
17	6.92	2766.0	0.1755
18	6.98	2788.9	0.1337
19	6.98	2790.3	0.1456
20	6.99	2796.2	0.1236

No.	(ppm)	(Hz)	Height
21	7.00	2797.6	0.1182
22	7.16	2861.3	0.0857
23	7.16	2862.6	0.0786
24	7.18	2869.0	0.1243
25	7.18	2870.4	0.1199
26	7.20	2876.8	0.0662
27	7.20	2878.2	0.0657
28	7.25	2898.8	0.2141
29	7.37	2946.4	0.2608
30	7.39	2953.3	0.3711

No.	(ppm)	(Hz)	Height
31	7.39	2954.7	0.3563
32	7.40	2959.7	0.0522
33	7.41	2962.5	0.1691
34	7.43	2969.8	0.1310
35	7.46	2984.0	0.2779
36	7.48	2991.3	0.3141
37	10.05	4018.0	0.1304

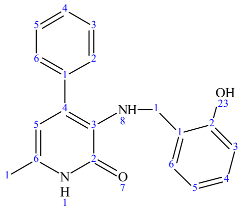

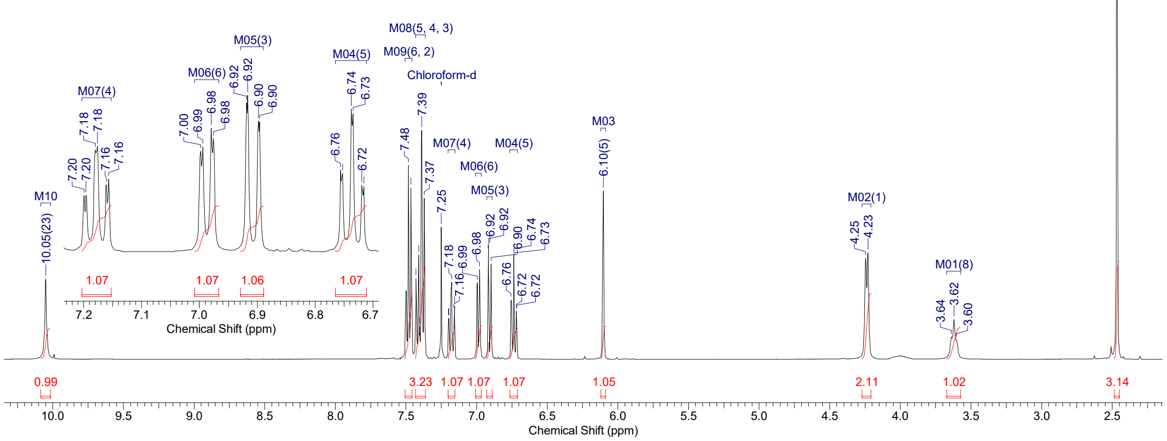

Figure S19. 3-((2-Hydroxybenzyl)amino)-6-methyl-4-phenylpyridin-2(1*H*)-one (3b)

^{13}C NMR (100 MHz).

No.	Atom	Exp. Shift (ppm)	No.	Atom	Exp. Shift (ppm)	No.	Atom	Exp. Shift (ppm)
1	1	18.59	8	3	127.84	15	4	137.21
2	1	48.81	9	4	128.55	16	1	137.44
3	5	109.49	10	6	129.18	17	6	140.94
4	3	116.66	11	2	129.18	18	2	157.47
5	5	118.48	12	6	129.26	19	2	162.67
6	4	124.36	13	1	129.26			
7	5	127.84	14	3	131.76			

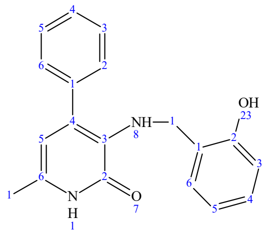

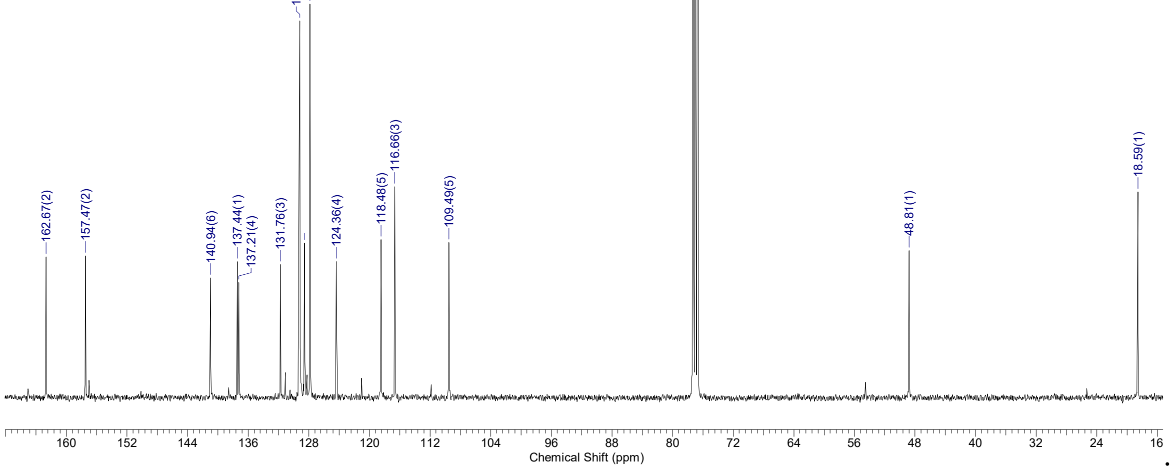

Figure S20. 3-((4-Methoxybenzyl)amino)-6-methyl-4-phenylpyridin-2(1*H*)-one (3c)

^{1}HNMR (400 MHz, $CDCl_3$).

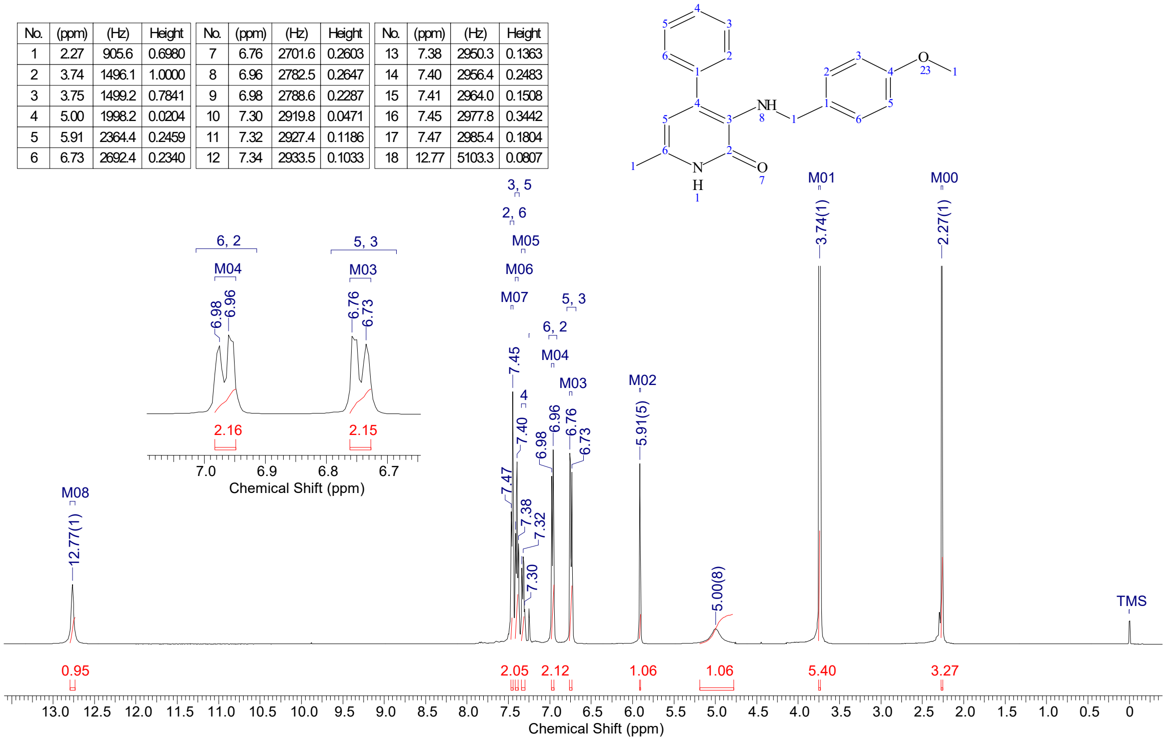

No.	(ppm)	(Hz)	Height
1	2.27	905.6	0.6980
2	3.74	1496.1	1.0000
3	3.75	1499.2	0.7841
4	5.00	1998.2	0.0204
5	5.91	2364.4	0.2459
6	6.73	2692.4	0.2340
7	6.76	2701.6	0.2603
8	6.96	2782.5	0.2647
9	6.98	2788.6	0.2287
10	7.30	2919.8	0.0471
11	7.32	2927.4	0.1186
12	7.34	2933.5	0.1033
13	7.38	2950.3	0.1363
14	7.40	2956.4	0.2483
15	7.41	2964.0	0.1508
16	7.45	2977.8	0.3442
17	7.47	2985.4	0.1804
18	12.77	5103.3	0.0807

Figure S21. 3-((4-Methoxybenzyl)amino)-6-methyl-4-phenylpyridin-2(1*H*)-one (3c)

^{13}C NMR (100 MHz).

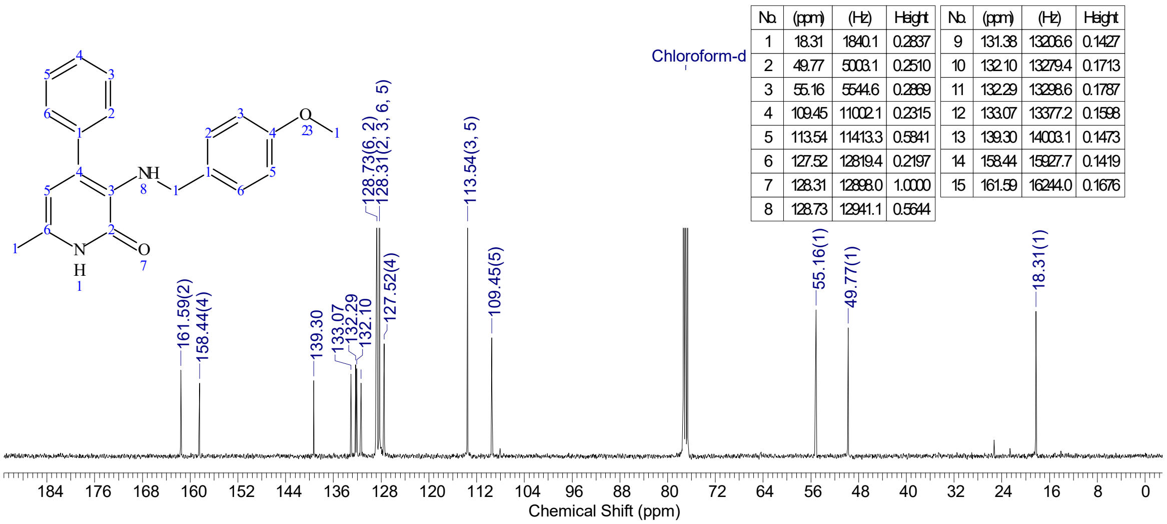

No.	(ppm)	(Hz)	Height
1	18.31	1840.1	0.2837
2	49.77	5003.1	0.2510
3	55.16	5544.6	0.2869
4	109.45	11002.1	0.2315
5	113.54	11413.3	0.5841
6	127.52	12819.4	0.2197
7	128.31	12898.0	1.0000
8	128.73	12941.1	0.5644
9	131.38	13206.6	0.1427
10	132.10	13279.4	0.1713
11	132.29	13298.6	0.1787
12	133.07	13377.2	0.1598
13	139.30	14003.1	0.1473
14	158.44	15927.7	0.1419
15	161.59	16244.0	0.1676

Figure S22. 6-Methyl-3-((3-nitrobenzyl)amino)-4-phenylpyridin-2(1*H*)-one (3d)

^{1}H NMR (400 MHz, $CDCl_3$).

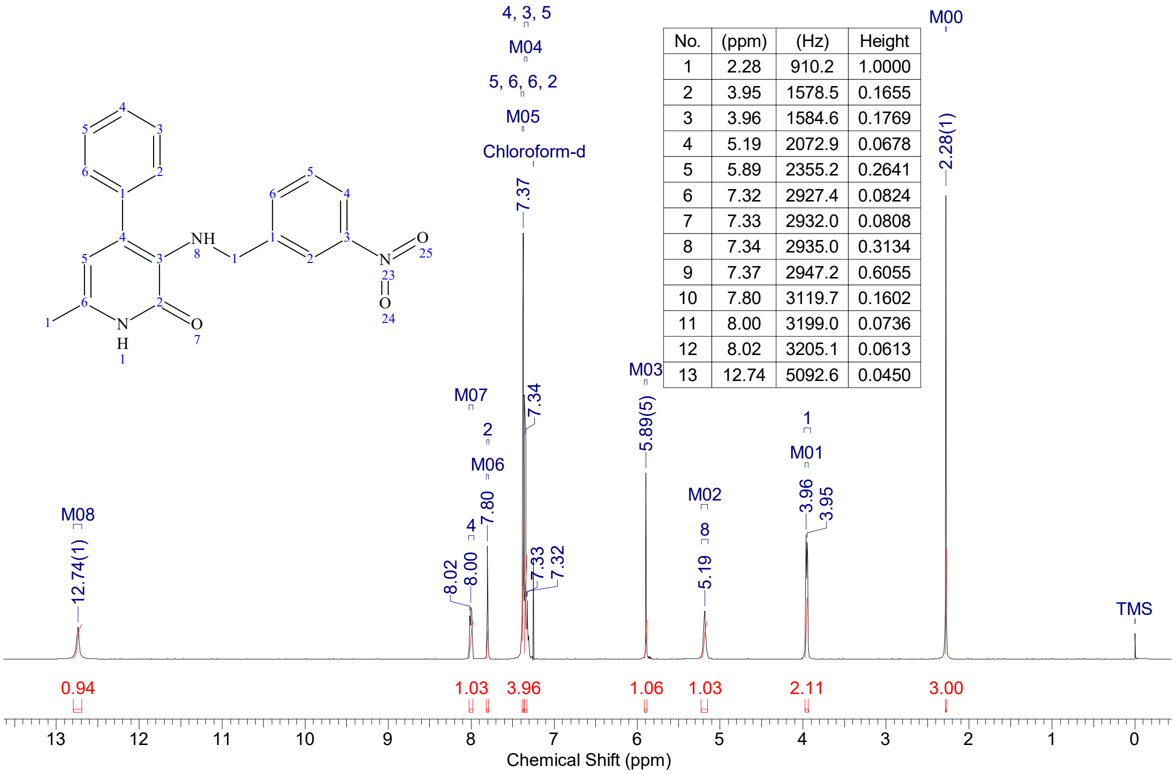

No.	(ppm)	(Hz)	Height
1	2.28	910.2	1.0000
2	3.95	1578.5	0.1655
3	3.96	1584.6	0.1769
4	5.19	2072.9	0.0678
5	5.89	2355.2	0.2641
6	7.32	2927.4	0.0824
7	7.33	2932.0	0.0808
8	7.34	2935.0	0.3134
9	7.37	2947.2	0.6055
10	7.80	3119.7	0.1602
11	8.00	3199.0	0.0736
12	8.02	3205.1	0.0613
13	12.74	5092.6	0.0450

Figure S23. 6-Methyl-3-((3-nitrobenzyl)amino)-4-phenylpyridin-2(1*H*)-one (3d)

^{13}C NMR (100 MHz).

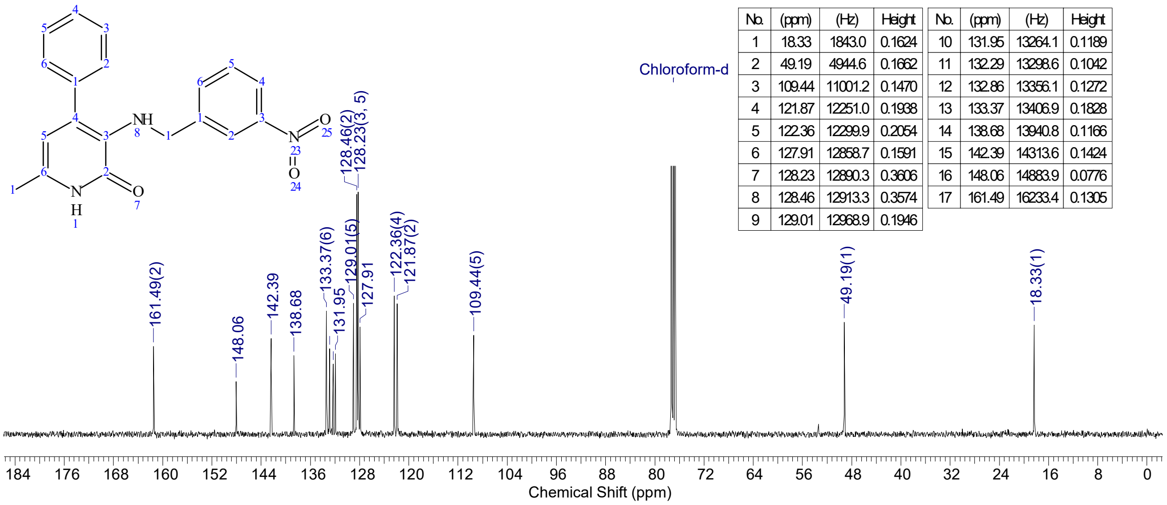

No.	(ppm)	(Hz)	Height	No.	(ppm)	(Hz)	Height
1	18.33	1843.0	0.1624	10	131.95	13264.1	0.1189
2	49.19	4944.6	0.1662	11	132.29	13298.6	0.1042
3	109.44	11001.2	0.1470	12	132.86	13356.1	0.1272
4	121.87	12251.0	0.1938	13	133.37	13406.9	0.1828
5	122.36	12299.9	0.2054	14	138.68	13940.8	0.1166
6	127.91	12858.7	0.1591	15	142.39	14313.6	0.1424
7	128.23	12890.3	0.3606	16	148.06	14883.9	0.0776
8	128.46	12913.3	0.3574	17	161.49	16233.4	0.1305
9	129.01	12968.9	0.1946				

Figure S24. 3-((3,4-Dimethoxybenzyl)amino)-6-methyl-4-phenylpyridin-2(1*H*)-one (3e)

^{1}H NMR (400 MHz, $CDCl_3$).

No.	(ppm)	(Hz)	Height	No.	(ppm)	(Hz)	Height	No.	(ppm)	(Hz)	Height
1	2.26	904.3	0.6596	8	6.56	2624.5	0.2977	15	7.37	2947.3	0.0870
2	2.27	906.7	0.0987	9	6.58	2631.9	0.1079	16	7.39	2955.9	0.2011
3	3.75	1499.7	0.3053	10	6.69	2674.7	0.1218	17	7.41	2963.2	0.1135
4	3.78	1510.7	1.0000	11	6.71	2682.0	0.1058	18	7.43	2971.8	0.2072
5	3.82	1525.4	0.6968	12	7.30	2919.2	0.0325	19	7.45	2979.1	0.1322
6	5.03	2012.0	0.0318	13	7.32	2927.8	0.0259	20	12.74	5091.9	0.0462
7	5.90	2359.2	0.1993	14	7.34	2933.9	0.0680				

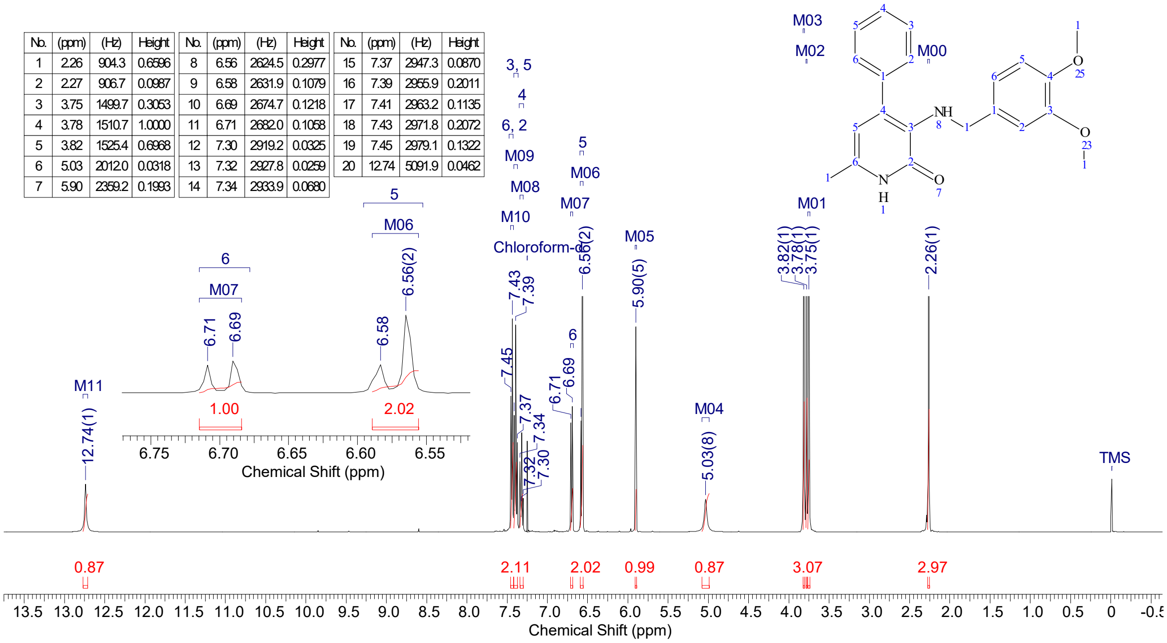

Figure S25. 3-((3,4-Dimethoxybenzyl)amino)-6-methyl-4-phenylpyridin-2(1*H*)-one (3e)

^{13}C NMR (100 MHz).

No.	(ppm)	(Hz)	Height	No.	(ppm)	(Hz)	Height	No.	(ppm)	(Hz)	Height
1	18.30	1839.2	0.2401	8	119.55	12018.1	0.2746	15	132.99	13368.6	0.1722
2	50.07	5033.7	0.2097	9	127.53	12820.3	0.1861	16	139.23	13996.4	0.1297
3	55.63	5592.5	0.2197	10	128.27	12894.1	0.4805	17	147.73	14850.4	0.1429
4	55.78	5606.9	0.2153	11	128.32	12899.9	0.4547	18	148.57	14934.7	0.1658
5	109.43	11000.2	0.1920	12	131.59	13227.7	0.1488	19	161.59	16244.0	0.1498
6	110.70	11127.7	0.2636	13	132.13	13282.3	0.1820				
7	110.74	11132.5	0.2827	14	132.75	13344.6	0.1782				

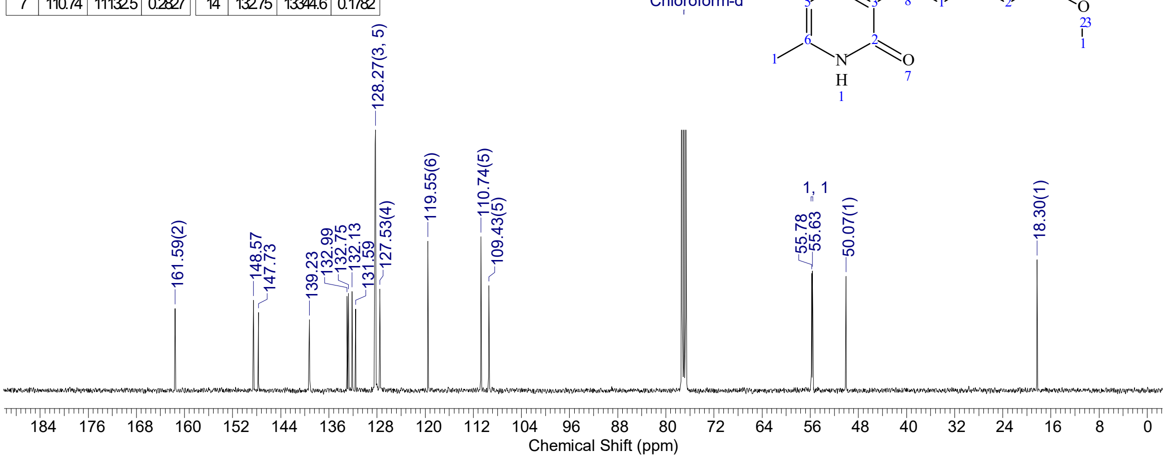

Figure S26. 3-((2,4-Dimethoxybenzyl)amino)-6-methyl-4-phenylpyridin-2(1*H*)-one (3f)

^{1}HNMR (400 MHz, $CDCl_3$).

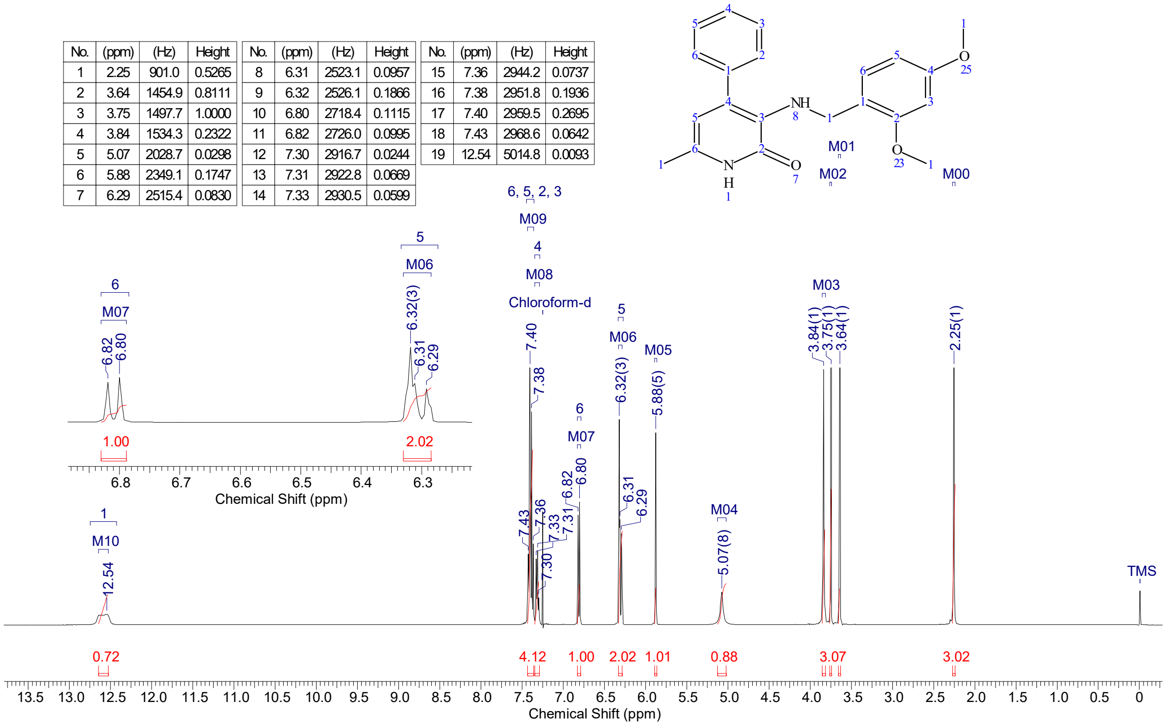

No.	(ppm)	(Hz)	Height	No.	(ppm)	(Hz)	Height	No.	(ppm)	(Hz)	Height
1	2.25	901.0	0.5265	8	6.31	2523.1	0.0957	15	7.36	2944.2	0.0737
2	3.64	1454.9	0.8111	9	6.32	2526.1	0.1866	16	7.38	2951.8	0.1936
3	3.75	1497.7	1.0000	10	6.80	2718.4	0.1115	17	7.40	2959.5	0.2695
4	3.84	1534.3	0.2322	11	6.82	2726.0	0.0995	18	7.43	2968.6	0.0642
5	5.07	2028.7	0.0298	12	7.30	2916.7	0.0244	19	12.54	5014.8	0.0093
6	5.88	2349.1	0.1747	13	7.31	2922.8	0.0669				
7	6.29	2515.4	0.0830	14	7.33	2930.5	0.0599				

Figure S27. 3-((2,4-Dimethoxybenzyl)amino)-6-methyl-4-phenylpyridin-2(1*H*)-one (3f)

^{13}C NMR(100 MHz).

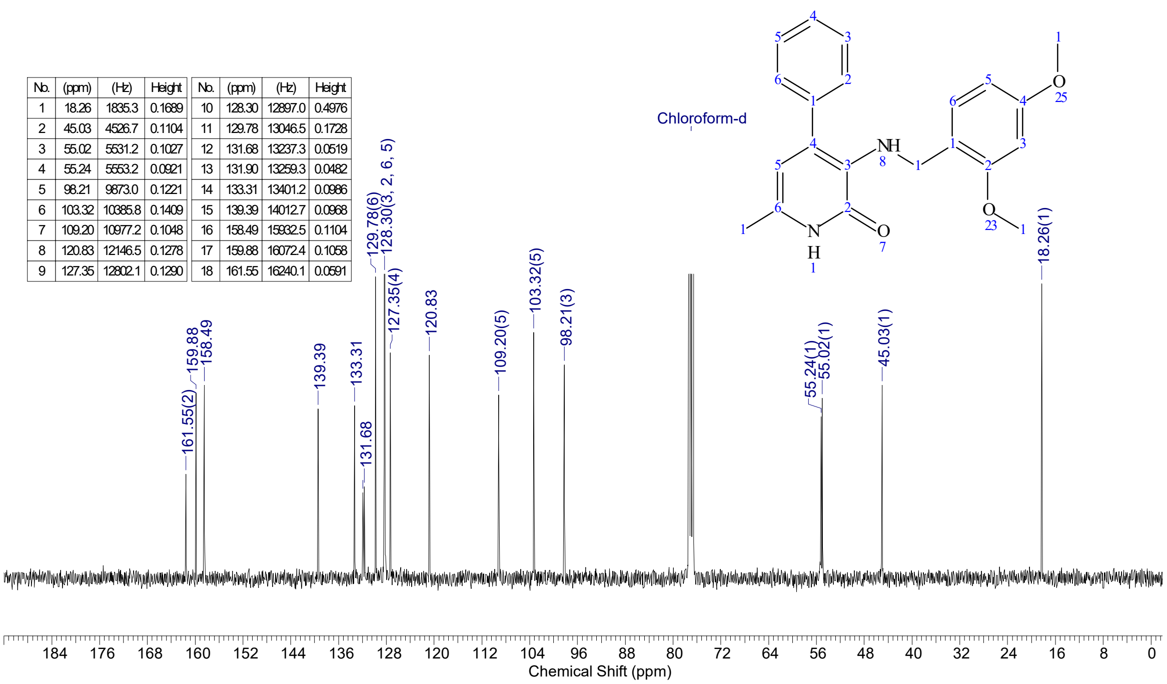

No.	(ppm)	(Hz)	Height	No.	(ppm)	(Hz)	Height
1	18.26	1835.3	0.1689	10	128.30	12897.0	0.4976
2	45.03	4526.7	0.1104	11	129.78	13046.5	0.1728
3	55.02	5531.2	0.1027	12	131.68	13237.3	0.0519
4	55.24	5553.2	0.0921	13	131.90	13259.3	0.0482
5	98.21	9873.0	0.1221	14	133.31	13401.2	0.0986
6	103.32	10385.8	0.1409	15	139.39	14012.7	0.0968
7	109.20	10977.2	0.1048	16	158.49	15932.5	0.1104
8	120.83	12146.5	0.1278	17	159.88	16072.4	0.1058
9	127.35	12802.1	0.1290	18	161.55	16240.1	0.0591

Figure S28. 3-((5-Bromo-2-hydroxybenzyl)amino)-6-methyl-4-phenylpyridin-2(1*H*)-one (3g) ^{1}HNMR (400 MHz, DMSO-d6).

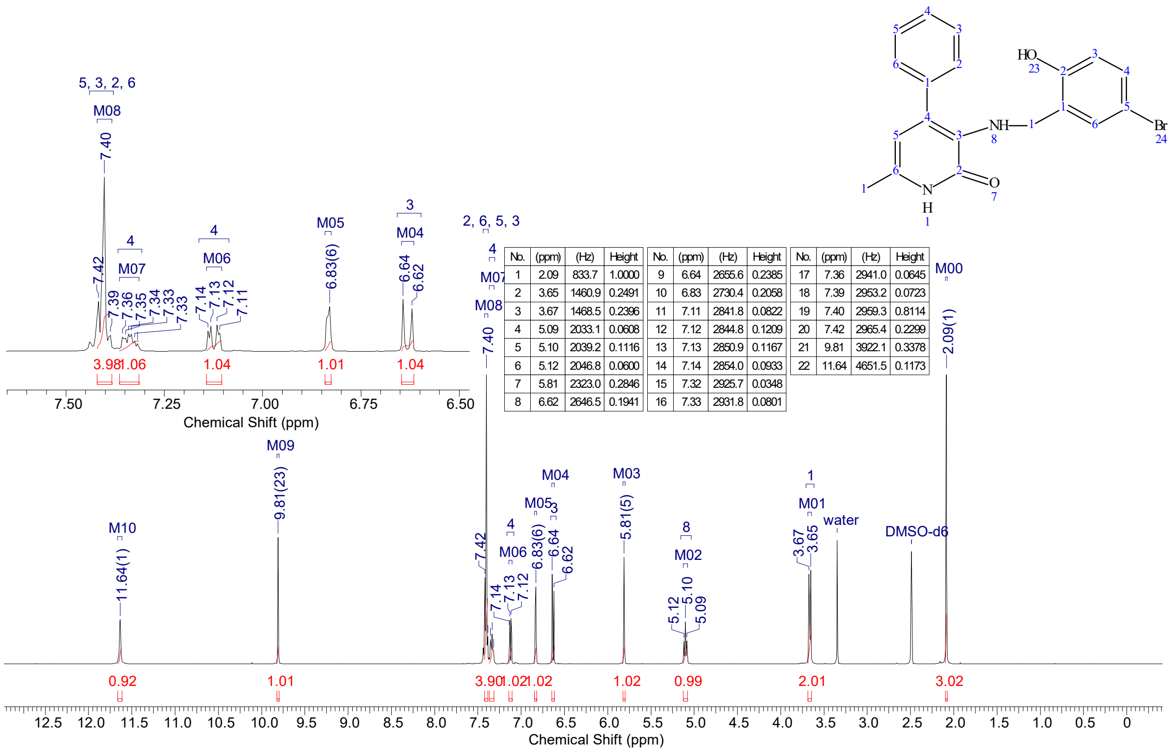

No.	(ppm)	(Hz)	Height	No.	(ppm)	(Hz)	Height	No.	(ppm)	(Hz)	Height
1	2.09	833.7	1.0000	9	6.64	2655.6	0.2385	17	7.36	2941.0	0.0645
2	3.65	1460.9	0.2491	10	6.83	2730.4	0.2058	18	7.39	2953.2	0.0723
3	3.67	1468.5	0.2396	11	7.11	2841.8	0.0822	19	7.40	2959.3	0.8114
4	5.09	2033.1	0.0608	12	7.12	2844.8	0.1209	20	7.42	2965.4	0.2299
5	5.10	2039.2	0.1116	13	7.13	2850.9	0.1167	21	9.81	3922.1	0.3378
6	5.12	2046.8	0.0600	14	7.14	2854.0	0.0933	22	11.64	4651.5	0.1173
7	5.81	2323.0	0.2846	15	7.32	2925.7	0.0348				
8	6.62	2646.5	0.1941	16	7.33	2931.8	0.0801				

Figure S29. 3-((5-Bromo-2-hydroxybenzyl)amino)-6-methyl-4-phenylpyridin-2(1*H*)-one (3g) ^{13}C NMR (100 MHz).

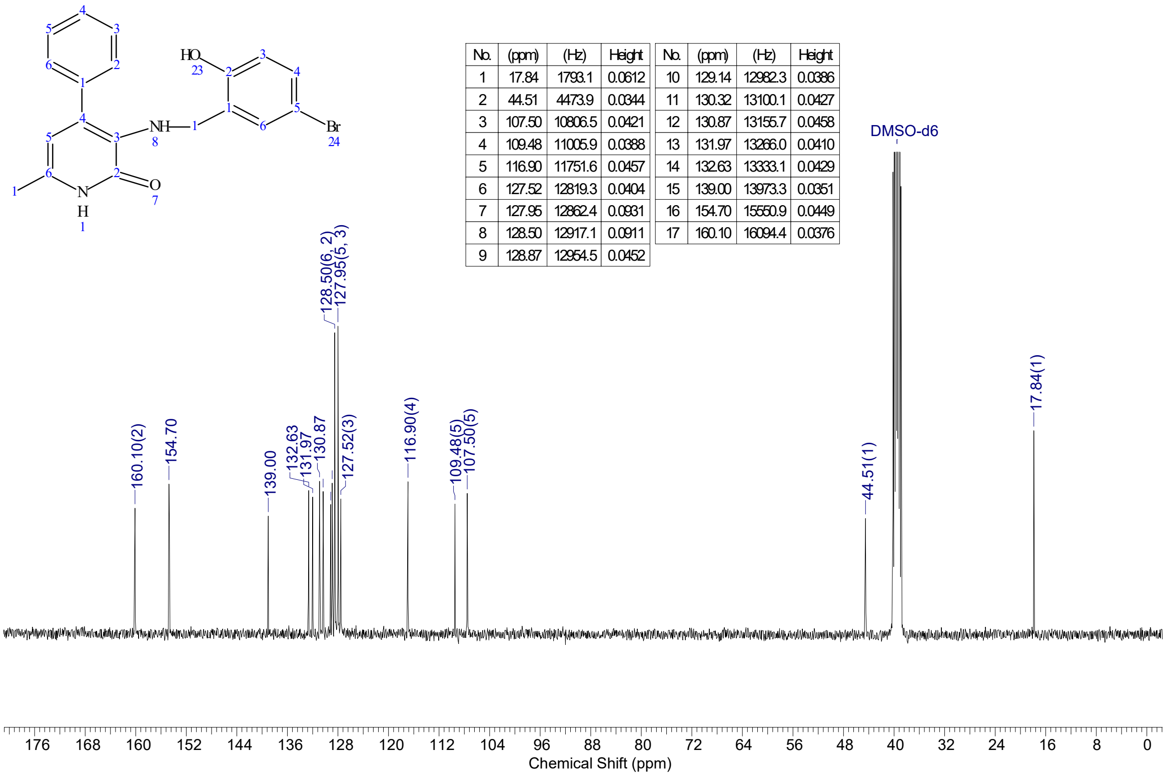

No.	(ppm)	(Hz)	Height
1	17.84	1793.1	0.0612
2	44.51	4473.9	0.0344
3	107.50	10806.5	0.0421
4	109.48	11005.9	0.0388
5	116.90	11751.6	0.0457
6	127.52	12819.3	0.0404
7	127.95	12862.4	0.0931
8	128.50	12917.1	0.0911
9	128.87	12954.5	0.0452

No.	(ppm)	(Hz)	Height
10	129.14	12982.3	0.0386
11	130.32	13100.1	0.0427
12	130.87	13155.7	0.0458
13	131.97	13266.0	0.0410
14	132.63	13333.1	0.0429
15	139.00	13973.3	0.0351
16	154.70	15550.9	0.0449
17	160.10	16094.4	0.0376

Figure S30. 3-((4-Dimethylaminobenzyl)amino)-6-methyl-4-phenylpyridin-2(1*H*)-one (3h) ^{1}HNMR (400 MHz, $CDCl_3$).

No.	(ppm)	(Hz)	Height	No.	(ppm)	(Hz)	Height	No.	(ppm)	(Hz)	Height
1	2.27	908.7	0.2738	7	6.63	2649.4	0.1094	13	7.39	2955.8	0.0445
2	2.89	1156.5	1.0000	8	6.95	2778.2	0.0974	14	7.41	2963.1	0.0962
3	3.70	1478.1	0.0822	9	6.97	2786.7	0.0792	15	7.43	2970.4	0.0531
4	4.97	1985.9	0.0214	10	7.30	2920.4	0.0165	16	7.49	2992.4	0.0879
5	5.93	2369.2	0.0825	11	7.32	2927.7	0.0441	17	7.50	2999.7	0.0664
6	6.60	2640.2	0.0913	12	7.34	2935.0	0.0371	18	12.83	5129.3	0.0142

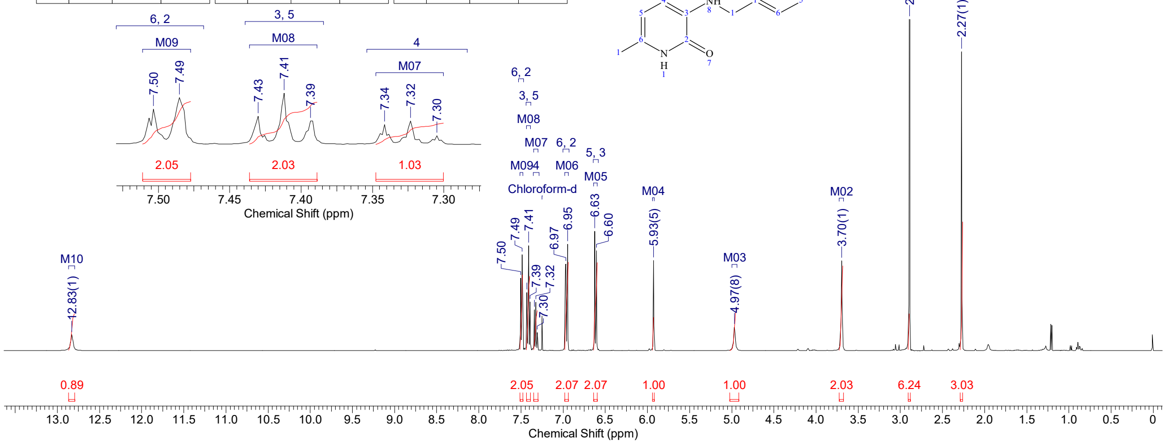

Figure S31. 3-((4-Dimethylaminobenzyl)amino)-6-methyl-4-phenylpyridin-2(1*H*)-one (3h) ^{13}C NMR (100 MHz).

No.	(ppm)	(Hz)	Height	No.	(ppm)	(Hz)	Height
1	18.31	1841.1	0.4164	9	128.34	12901.8	0.7771
2	40.69	4090.6	0.7972	10	128.53	12921.0	0.9577
3	50.04	5029.9	0.3744	11	131.05	13174.0	0.2194
4	109.43	11000.2	0.3265	12	131.90	13259.3	0.2954
5	112.51	11309.8	1.0000	13	133.41	13410.7	0.2704
6	127.40	12806.9	0.3457	14	139.49	14022.2	0.2299
7	128.20	12887.4	0.3761	15	149.65	15044.0	0.2260
8	128.26	12893.2	0.8446	16	161.63	16247.8	0.2595

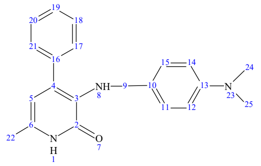

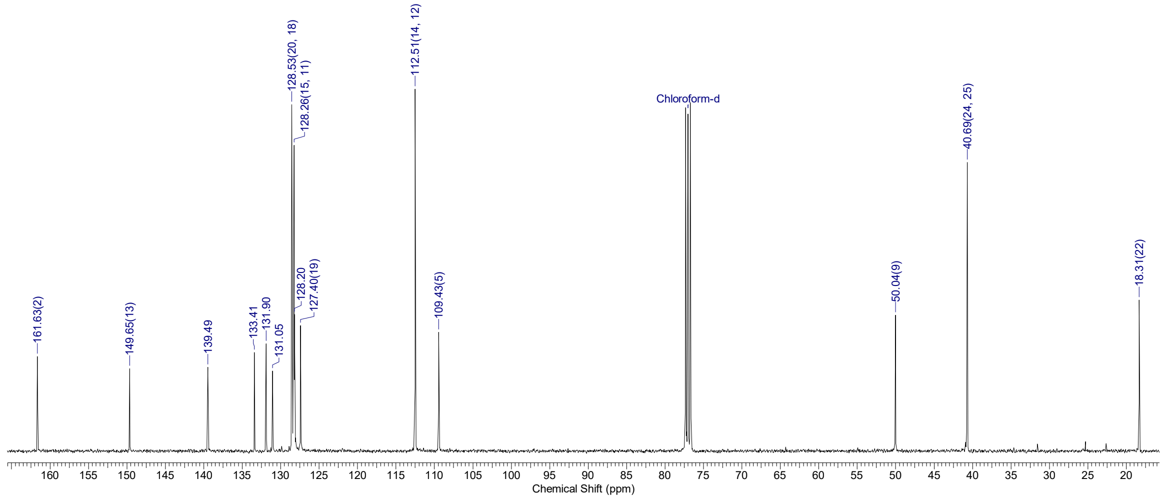

Supplement: Supplementary file 1 [file molecules-27-05362-s001.zip › molecules-1874577-supplementary.pdf]
